# Supplementary material for: The Structure of the PanD/PanZ Protein Complex Reveals Negative Feedback Regulation of Pantothenate Biosynthesis by Coenzyme A
Source: Chem Biol. 2015 Apr 23;22(4):492–503. doi: 10.1016/j.chembiol.2015.03.017 (PMC4410942; doi:10.1016/j.chembiol.2015.03.017)
Supplement: Document S1. Figures S1–S7, Table S1, and Supplemental Materials and Methods [file mmc1.pdf]

Chemistry & Biology, Volume *22*

Supplemental Information

The Structure of the PanD/PanZ Protein Complex Reveals Negative Feedback Regulation of Pantothenate Biosynthesis by Coenzyme A

Diana C.F. Monteiro, Vijay Patel, Christopher P. Bartlett, Shingo Nozaki, Thomas D. Grant, James A. Gowdy, Gary S. Thompson, Arnout P. Kalverda, Edward H. Snell, Hironori Niki, Arwen R. Pearson, and Michael E. Webb

Supplemental figures

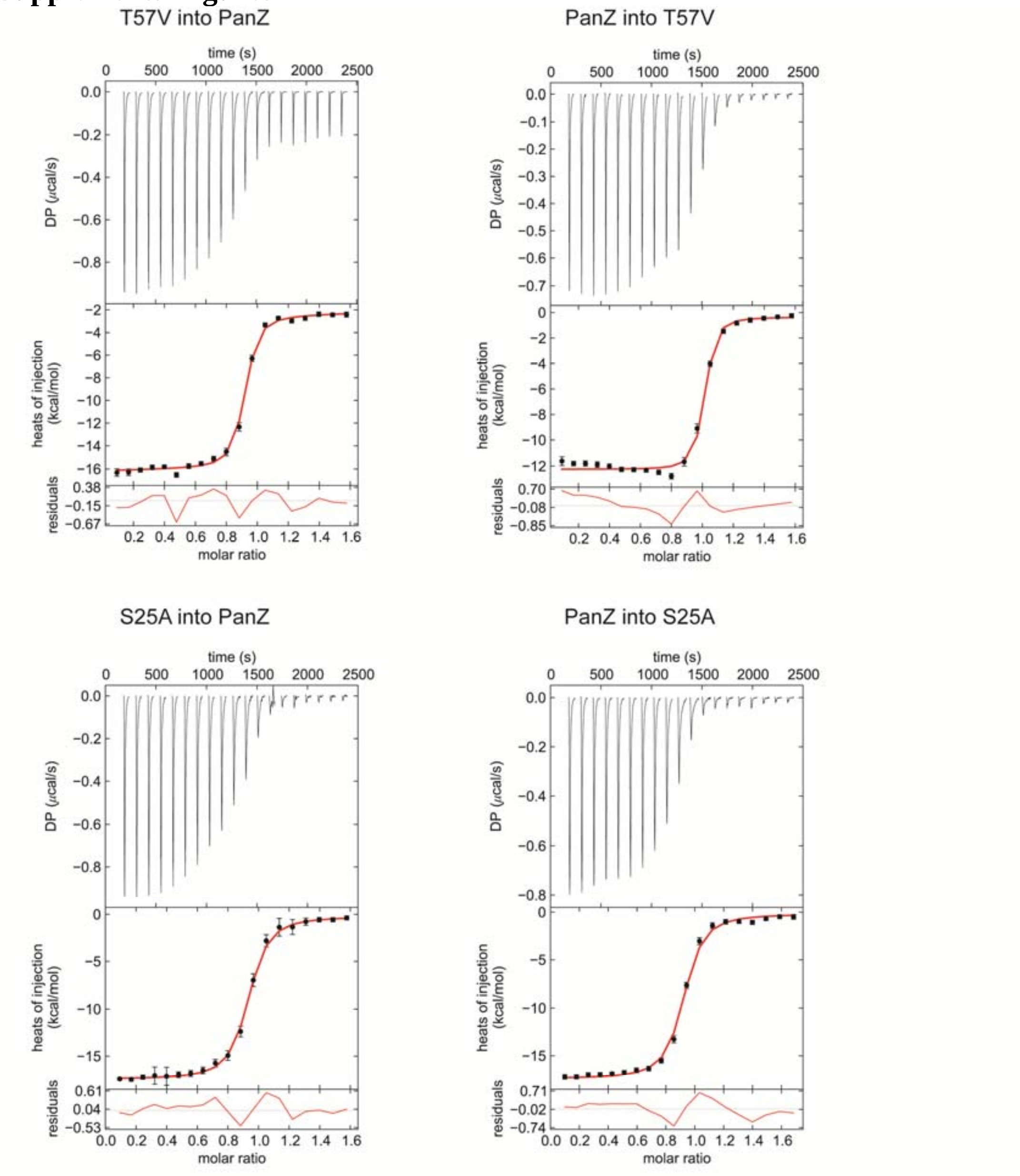

Mutant	Log K_a	K_d /nM	ΔH /kcal mol^{-1}	ΔG /kcal mol^{-1}	ΔS /cal mol^{-1} K^{-1}
T57V	7.457 ± 0.045	34.9 ± 3.6	-11.89 ± 0.07	-10.16	-5.8
S25A	6.803 ± 0.015	157.2 ± 5.5	-17.20 ± 0.03	-9.27	-26.6

Figure S1, related to figures 2 and 4 Global analysis of interaction between PanZ and PanD by ITC. **A** Pairwise titration of 263 μM PanD(T57V) into 35 μM PanZ in the presence of 400 μM AcCoA and 263 μM PanZ into 35 μM PanD(T57V) in the presence of 400 μM AcCoA. **B** Pairwise titration of 257 μM PanD(S25A) into 32 μM PanZ in the presence of 394 μM AcCoA and 263 μM PanZ into 35 μM PanD(S25A) in the presence of 400 μM AcCoA. **C** Thermodynamic parameters for interaction obtained by global fitting of both sets of pairwise titrations. Quoted errors are standard errors (n=38) based on direct determination of the parameter values corresponding to critical changes in χ^2 corresponding to a 66% confidence interval.

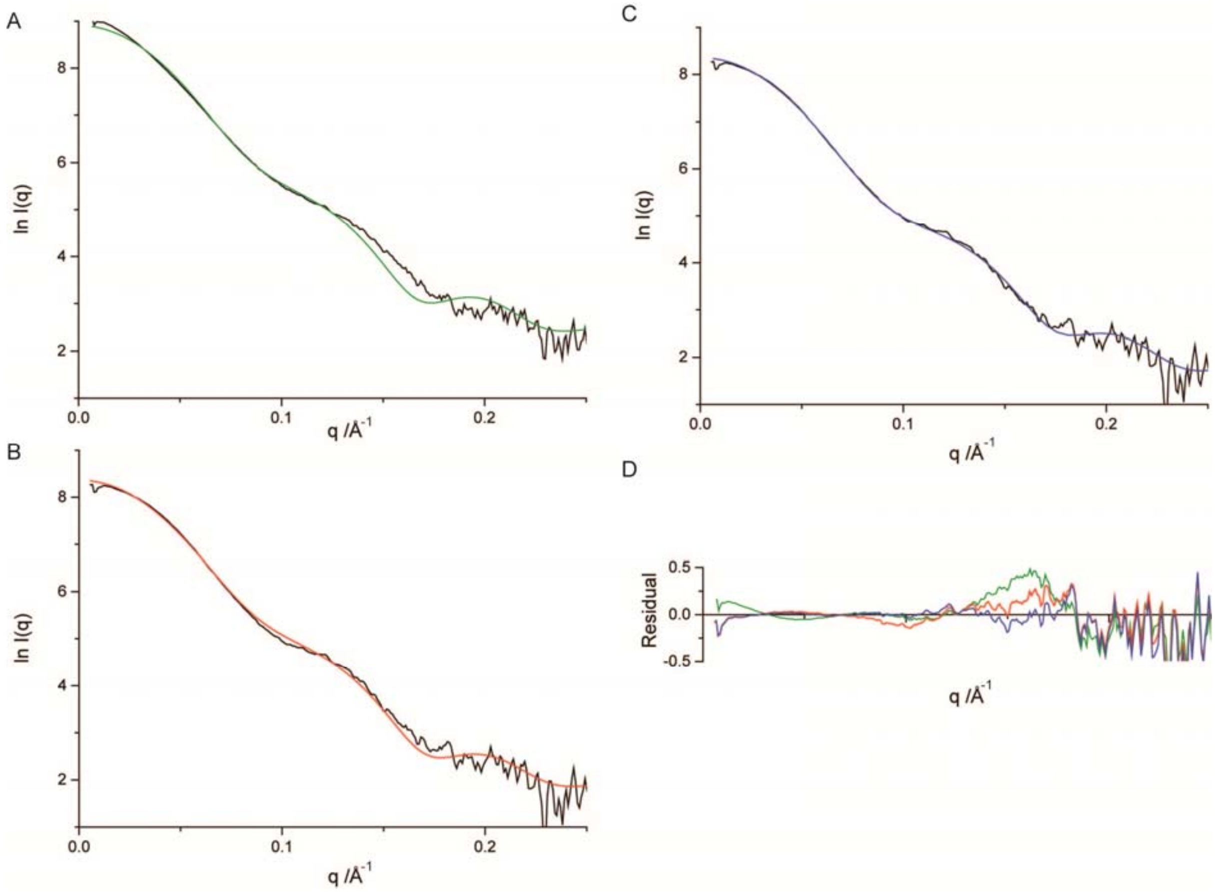

Figure S2, related to figure 2 SAXS data and fitting. **A** Raw SAXS data for the PanD(T57V)-PanZ.AcCoA complex (black) compared with predicted data for the crystallographically resolved heterooctameric complex (green). **B** Inclusion of a population of dimers of heterooctamers leads to an improved fit (red) compared to the monomer. **C** Subsequent inclusion of the eight *C*- and *N*-terminal affinity purification tags using a coarse-grained model leads to a further improved fit (blue). **D** Residuals from three sequential rounds of data fitting: heterooctamer (green), inclusion of dimer of heterooctamers (red), inclusion of affinity tags (blue).

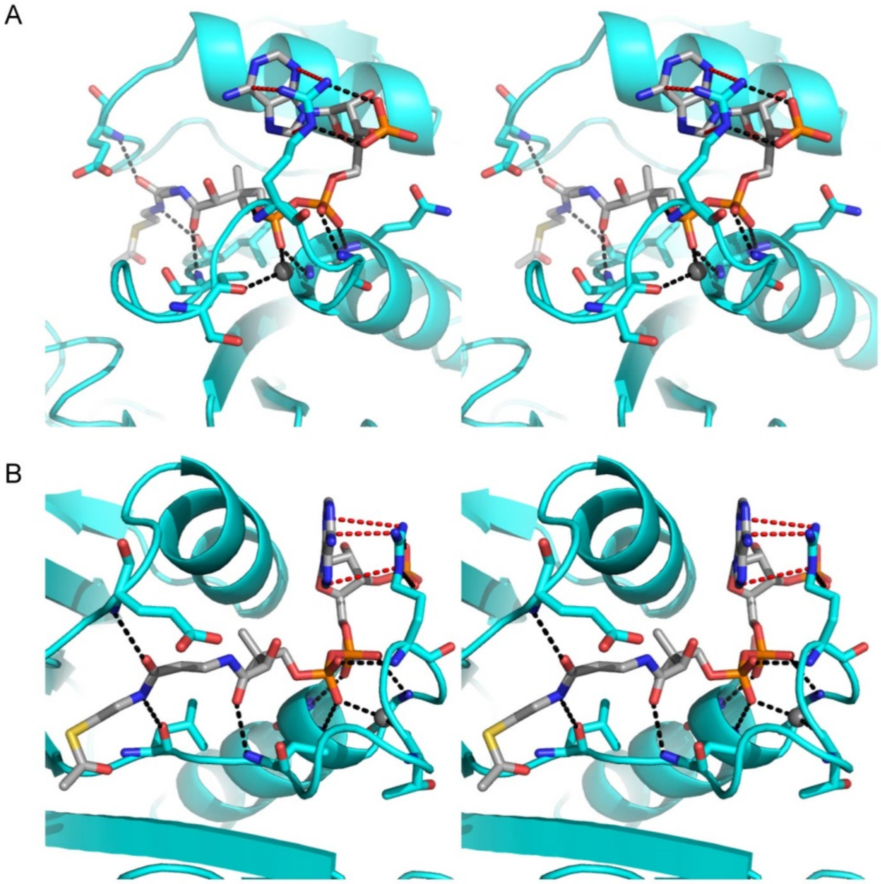

Figure S3, related to figure 3 Hydrogen-bonding interactions in the PanZ AcCoA binding pocket. **A** Front view of the AcCoA binding pocket (in stereo), showing hydrogen-bonds (black dashed lines) and cation-π interactions (red dashed lines) between AcCoA, Mg^{2+} and PanZ. All interacting residues, with the exception of Glu23, Gly78 and Gln79 are located in the PanZ P-loop. **B** Side view of the same binding pocket and interactions (also in stereo).

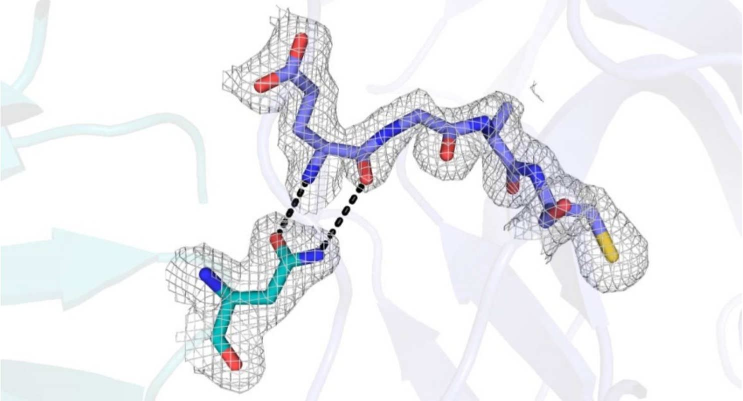

Figure S4, related to figure 5

Sample electron density for the PanD(S25A).PanZ.AcCoA complex showing conformation of the activatable loop and bidentate hydrogen bonding interactions between PanZ-Asn45 and the backbone amide of PanD-Glu23. The $2F_0$-F_c electron density map is contoured at 1 r.m.s.d.

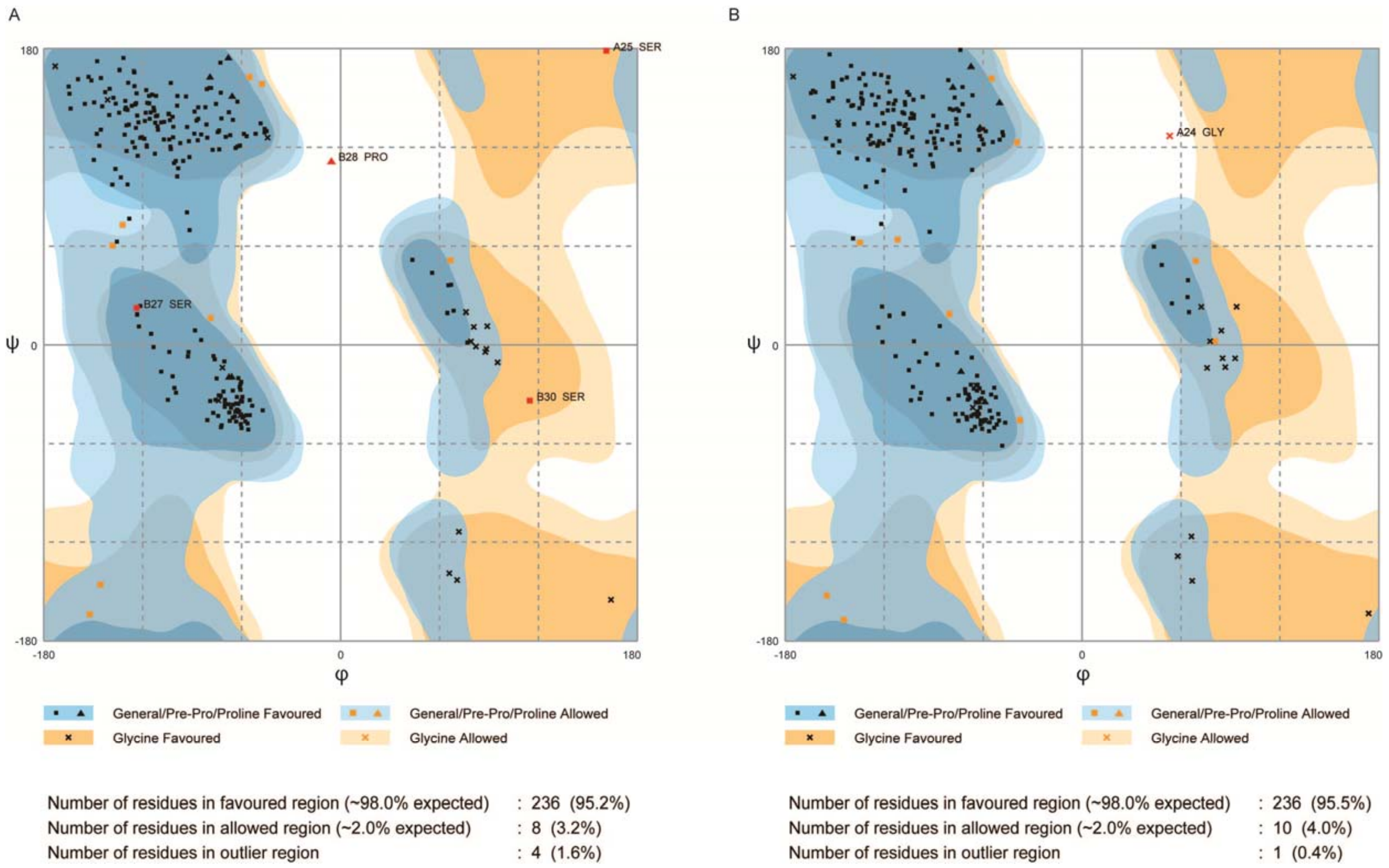

Figure S5, related to figure 5 Ramachandran plots for the PanD-PanZ complex. **A** Ramachandran plot for the PanD(T57V)-PanZ.AcCoA complex. Residue PanD-Ser25 (A25 SER) lies in a outlier region. (Residues B27-B30 correspond to a poorly defined region of PanZ) **B** Ramachandran plot for the PanD(S25A)-PanZ.AcCoA complex. Residue PanD-Gly24 lies in an outlier region (Lovell et al., 2003). Ramachandran plots were generated using RAMPAGE.

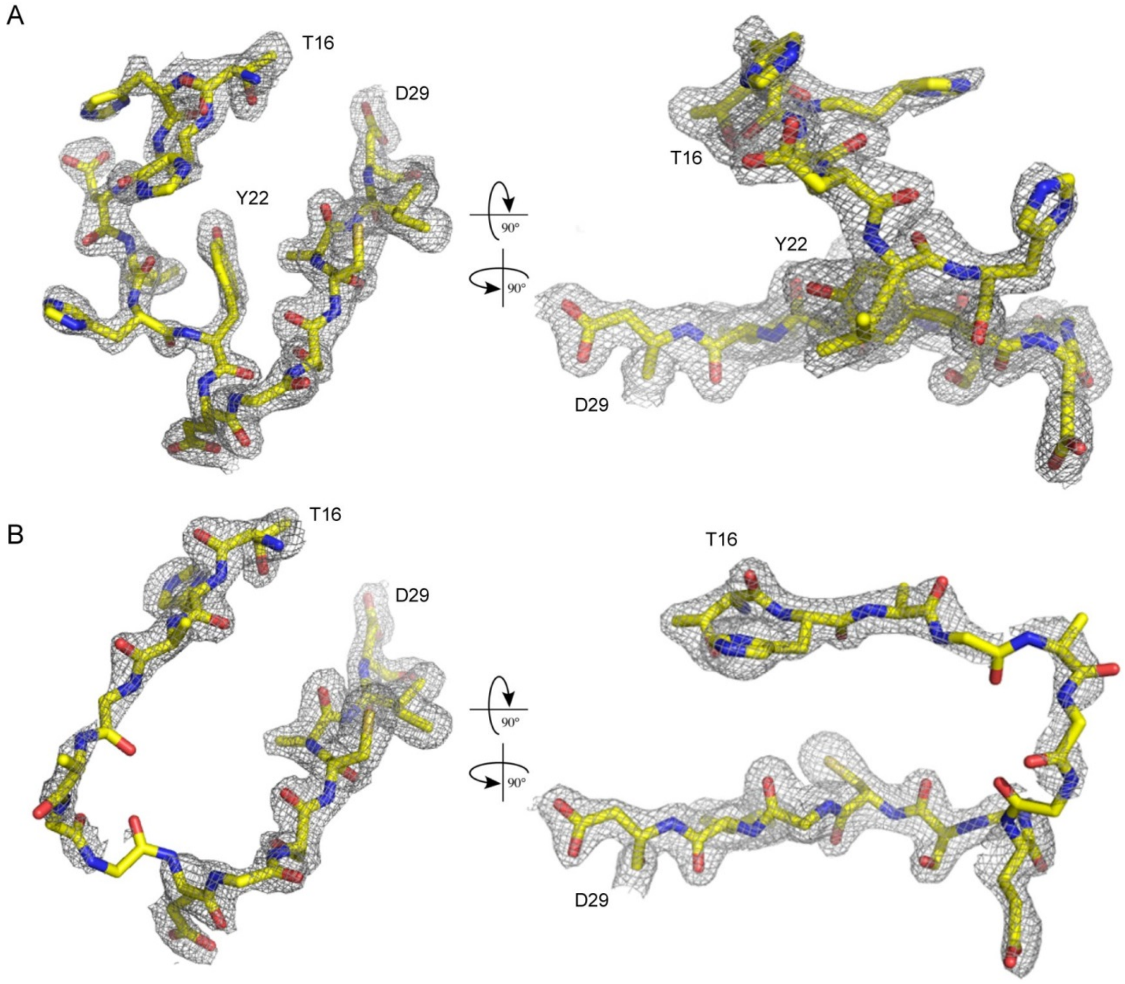

Figure S6, related to figure 5 and 7 The different processing loop conformations adopted by the two pADC zymogen protomers in the asymmetric unit of the structure solved previously by Schmitzberger *et al.* (PDB 1PPY (Schmitzberger et al., 2003)) Residues Thr16-Asp29 are represented as sticks and the accompanying $2F_0$-F_c electron density map is contoured at 1 r.m.s.d. **A** The non-activatable conformation observed in conformer A, showing a well defined electron density map throughout the entire loop. **B** The activatable conformation observed in protomer B, showing a much less well defined electron density map in the processing region, with the sidechain of Tyr22 unresolved. In the absence of PanZ, this conformation is disfavoured and only the electron-density for residues Thr16-Ala18 (which form a new β-sheet) is well defined. Consequently once the β-sheet has formed the remainder of the loop does not adopt one single conformation, explaining the poorly defined electron density.

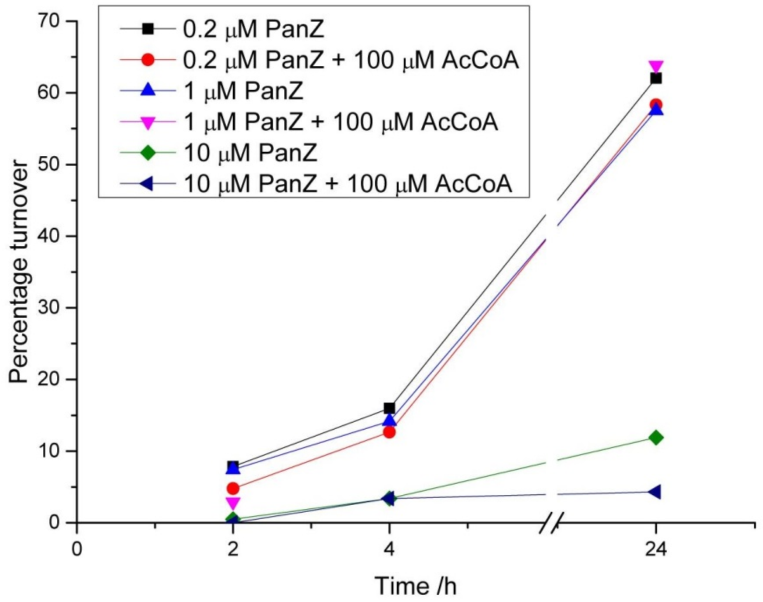

Figure S7, related to figure 6 ADC inhibition by PanZ. The percentage turnover of L-aspartate by ADC over time was monitored using 500 MHz ^{1}H NMR. Increase in the concentration of PanZ in solution leads to decreased turnover.

Table S1, related to figure 2 Data collection and refinement statistics (molecular replacement)

	pADC(T57V)-PanZ	pADC(S25A)-PanZ
**Data collection**		
Space group	I4	I4
Cell dimensions		
a, *b*, *c* (Å)	86.3, 86.3, 80.9	86.3, 86.3, 80.8
α, β, γ (°)	90.0, 90.0, 90.0	90.0, 90.0, 90.0
Resolution (Å)	33.7-1.70 (1.74-1.70)*	33.7-2.10 (2.16-2.10)*
R_{sym} or R_{merge}	3.7 (39.8)	18.1 (62.1)
R_{pim}	3.6 (36.6)	15.5 (51.7)
$I / \sigma I$	13.3 (2.2)	5.7 (1.8)
Completeness (%)	99.7 (99.7)	99.5 (99.8)
Redundancy	2.4 (2.3)	3.2 (3.1)
**Refinement**		
PDB ID	4CRZ	4CS0
Resolution (Å)	29.52 (1.70)	33.7 (2.10)
No. reflections	30609	16351
R_{work} / R_{free}	14.46 / 17.52	17.21 / 23.69
No. atoms		
Protein	2064	2021
Ligand/ion	65	62
Water	145	62
B-factors		
Protein (main chain)	20.35	24.29
Protein (side chain)	25.99	28.57
Ligand/ion	20.35	28.47
Water	32.45	26.86
R.m.s. deviations		
Bond lengths (Å)	0.023	0.019
Bond angles (°)	2.33	2.09

*All datasets were collected from a single crystal, values in parentheses are for highest-resolution shell.

Supplemental Materials and Methods:

Protein expression and purification

The coding sequence for C-terminally hexahis-tagged PanZ was subcloned into the pET28a backbone from pBAD24 by PCR to allow protein overexpression by auto-induction of T7 RNA polymerase. The coding sequence for PanZ, including the ribosome-binding site, was amplified by PCR using the primers PanZ-F (5′-GTGATGCTAGCAGGAGGAATTCC-3′) and PanZ-R (5′-GAATCTCGAGACCGCTACACTTCTCC-3′) from the pBAD24-PanZ construct described previously (Nozaki et al., 2012). The amplified product was digested (NheI and XhoI) before ligation into pET28a previously linearised with XbaI and XhoI. The sequence of the construct was confirmed by DNA sequencing (GATC).

His-tagged WT-ADC, PanD(T57V), PanD(S25A), PanZ and PanZ(N45A) were overexpressed using an *E. coli ΔpanD ΔpanZ* (DE3) cell strain as described previously (Monteiro et al., 2012; Nozaki et al., 2012). For ITC activity assays, His-tagged WT-ADC was overexpressed using *E. coli* C41 (DE3). His-tagged WT-ADC, PanD(T57V) and PanD(S25A) were overexpressed from the vectors pRSETA-ADC-WT (Saldanha et al., 2001), pRSETA-ADC(T57V) (Webb et al., 2014), pRSETA-ADC(S25A) (Schmitzberger et al., 2003) and pET28a-PanZ using an autoinduction protocol (Studier, 2005). For ITC kinetic analysis, ADC was overexpressed from pRSETA-ADC-WT in *E. coli* C41 (DE3). PanZ(N45A) was overexpressed from the vector pBAD24-PanZ(N45A) using arabinose induction as described previously (Nozaki et al., 2012). Cells were isolated by centrifugation (10,000*g*, 15 min), resuspended in buffer A (50 mM potassium phosphate, 300 mM NaCl, pH 7.4) containing 10 mM imidazole, mechanically lysed using a Constant Systems cell disrupter (20 kpsi) and the lysate cleared by centrifugation (30,000*g*, 45 min). DNase I (Roche, ~0.5 mgL^{-1} culture) was added to the cleared lysate before application to a Ni-NTA agarose (Qiagen) column under gravity flow. The column was washed with 10 column volumes of wash buffer (Buffer A + 50 mM imidazole) before elution of protein fractions using elution buffer (Buffer A + 250 mM imidazole). Protein-containing fractions, which were identified by SDS–PAGE, were combined and concentrated using centrifugal concentration (Amicon, 10 kDa MWCO). Protein was purified to apparent homogeneity by isocratic elution from either a Hi-Load 26/60 or 16/60 Superdex 75 column (GE) mounted on an Akta Purifier FPLC system in 50 mM Tris–HCl, 100 mM NaCl, 0.1 mM DTT, pH 7.4 at 4 ºC. Protein identity was confirmed by electrospray mass spectrometry using a Bruker HCT-Ultra LC–MS system. Protein concentrations were determined by UV absorption at 280 nm using theoretical estimates for the protein concentration based on the primary sequence of the protein (ProtParam (Gasteiger et al., 2005): PanD ε_{280} 15470 M^{-1} cm^{-1}, PanZ ε_{280} 26470 M^{-1} cm^{-1}).

Protein crystallization and structure solution

Solutions of the protein complexes were prepared using a 10:11 ratio of PanD(T57V) to PanZ. The proteins were then concentrated to a total protein concentration of 9-11 mg mL^{-1} using centrifugal concentration (Amicon, 10 kDa MWCO) and a two-fold molar excess of AcCoA added. Sparse matrix screens using Crystal Screen, Crystal Screen 2, Index, and Salt RX (Hampton Research), and Wizard I and II (Emerald Bioscience) were set up using an Oryx 6 Douglas crystallization robot (Douglas Instruments) in MRC 96-well plates

(Molecular Dimensions) with 1.0 µL protein:1.0 µL mother liquor drops at 18 ºC. Crystals were obtained in 20% (*w*/*v*) polyethylene glycol (PEG) 3350, 0.1 M bis-tris propane pH 7.4, 0.2 M potassium thiocyanate. Optimisation was carried out by pH and salt concentration variation and crystals grown using the hanging drop vapour diffusion method. Crystals were mounted on MicroMeshes™ (Mitegen) and data collected at RT (298 K).

The PanD(T57V)-PanZ complex and PanD(S25A)-PanZ complex were collected at room temperature using the in-house source (MicroMax-007 HF microfocus rotating anode generator, 30 mA), $\lambda = 1.5418$ Å. 60 frames of 1° oscillation, 30 s exposure and full transmission were collected with to a maximum resolution of 1.64 Å and 2.1 Å respectively. Data were integrated in spacegroup *I4* using iMosflm (Leslie and Powell, 2007), and scaled and merged using Aimless (Evans and Murshudov, 2013). Phasing was carried out by molecular replacement using Molrep (Vagin and Teplyakov, 1997). PDB 4AZD was placed first, followed by 2K5T. One protomer of each (PanD and PanZ) are found in the asymmetric unit and these form the heterooctameric complex by symmetry. The solution was checked by removing the CoA ligand and searching for the corresponding positive signal on the F_o-F_c difference electron density map after 20 cycles of restrained refinement. The solutions were subjected to iterative rounds of manual rebuilding and refinement using Coot (Emsley et al., 2010) and refinement with Refmac5 (Murshudov et al., 2011).

Isothermal titration calorimetry

Isothermal titration calorimetry experiments were performed using a Microcal iTC200 (GE) or Microcal VP-ITC (GE) thermostatted at 25 °C. For the microcal iTC200, the ligand sample was loaded into the sample cell (200 µL) and the titrant into the sample syringe (40 µL). Each titration series consisted of a 0.4 µL injection followed by 19 injections of 2 µL. For the VP-ITC, the ligand sample was loaded into the sample cell (1.5 mL) and the titrant into the sample syringe (300 µL). Each titration series consisted of a 2 µL injection followed by 29 injections of 10 µL each. Samples were prepared in 50 mM Tris–HCl, 100 mM NaCl, pH 7.4, 0.1 mM DTT. For titrations in the presence of excess AcCoA, this was added to both the ligand and titrant sample to the same final concentration without any attempt to account for copurified CoA in the PanZ sample. Data were analysed in Origin 6.5. After baseline substraction data were fitted to a single site-binding model. For global fitting, data were integrated using NITPIC (Keller et al., 2012) before global fitting to a one-site binding model in SEDPHAT (Houtman et al., 2007). Errors in parameters obtained through global fitting were estimated using parameter values corresponding to critical χ^2 values on computed error surface projections using SEDPHAT.

For kinetic experiments, isothermal titration calorimetry experiments were performed using a Microcal VP-ITC (GE) thermostatted at 25 °C. Samples were prepared in 50 mM potassium phosphate pH 7.0. The enzyme sample was loaded into the sample cell (2 mL) and the substrate (monosodium L-aspartate, 25 mM) into the sample syringe (200 µL). Each titration series consisted of a 25 injections of 2 µL. The reaction rate was estimated based on the baseline change after each injection (using the measured enthalpy of reaction of 3.8 kcal mol^{-1} as a conversion factor.). All measurements were taken relative to a linear extrapolation of the pre-titration baseline.

Complementation assays

Wild-type cells (MG1655) harboring vector plasmid (pBAD24), Δ*panZ* cells (MG1655 ΔpanZ) harboring vector plasmid and Δ*panZ* cells harboring pBAD24-*panZ-his* were incubated overnight in 3 mL of L broth containing 50 µg/mL of ampicillin at 37 ºC. Cells were washed with M9 salt solution (6 g/L Na_2HPO_4, 3 g/L KH_2PO_4, 0.5 g/L NaCl, 1 g/L NH_4Cl) and resuspended in M9 salt solution to an adjusted OD_{600} of each cell suspension of 1.0. Cell suspensions were serially diluted in M9 salt solution at 10^{-2}, 10^{-3}, 10^{-4} 10^{-5} and 10^{-6} and 3 µL of each dilution was spotted on M9 agar plates containing 0.2% glucose and 50 µg/mL of ampicillin, M9 agar plates containing 0.2% of glucose, 0.5 mM of β-alanine, and 50 µg/mL of ampicillin, M9 agar plates containing 0.2% of L-(+)-arabinose and 50 µg/mL of ampicillin, and M9 agar plates containing 0.2% of L-(+)-arabinose, 0.5 mM of β-alanine, and 50 µg/mL of ampicillin. The M9 glucose plates and M9 arabinose plates were incubated at 37 ºC for 24 hours and for 48 hours, respectively and observed for formation of colonies.

SAXS

Small angle X-ray scattering data were collected on beamline 4-2 of the Stanford Synchrotron Radiation Lightsource (SSRL). Data were collected at a wavelength of λ = 1.3Å for eight consecutive two second exposures from three concentrations of protein ranging from 0.20 to 2.9 mg/mL. PanD(T57V) and PanZ were mixed together with AcCoA in a 1:1:2 ratio, respectively. The mixture was concentrated to c. 10 mg/mL using centrifugal concentration (Amicon, 10 kDa MWCO). The flowthrough was used as the buffer blank (50 mM Tris–HCl, 100 mM NaCl, 0.1 mM DTT, pH 7.4) and subtracted from the total protein solution scattering. The data were integrated with SASTool and examined with PRIMUS (Konarev et al., 2003). Analysis of eight consecutive time frames showed that no radiation damage took place over the course of the experiment. The SAXS data for each concentration were investigated for aggregation using Guinier plots. Radius of gyration estimates were derived by the Guinier approximation $I(q) = I(0) \exp(-q^2R_g^2/3)$ with $qR_g < 1.3$, where $q = 4\pi\sin\theta/\lambda$ and were evaluated in PRIMUS. GNOM (Svergun, 1992) was used to compute the pair distribution function, $P(r)$, and to determine the maximum particle dimension. Chicken egg white lysozyme was used as a protein standard to estimate the molecular weight from $I(0)$ calculated by GNOM. A slight increase in particle R_g was detected as a function of concentration when analysing all three dilutions of the protein solution, suggesting the presence of a larger oligomer. Evaluation of various symmetry mates coupled with the use of the software program OLIGOMER identified a candidate for the larger oligomer as a dimer of the crystallographically determined complex of PanD(T57V)-PanZ. OLIGOMER estimated a volume fraction of 32% of the larger oligomer in the lowest concentration and 39% in the highest concentration. Therefore, a 39% contribution of the simulated scattering profile of the larger oligomer, computed by FoXS (Schneidman-Duhovny et al., 2013), was subtracted from the SAXS data of the highest concentration to yield the scattering from a single PanD(T57V)-PanZ complex molecule for further analysis. Ten *ab initio* shape reconstructions were generated by DAMMIF (Franke and Svergun, 2009) using P4 symmetry

and averaged with DAMAVER (Volkov and Svergun, 2003). CORAL (Petoukhov et al., 2012) was used determine the positions of the crystallographically disordered residues. SUPCOMB (Kozin and Svergun, 2001) was used to align the high-resolution model with the envelope reconstruction).

Small molecule NMR

Activated ADC was obtained via enzymatic activation. A 2:1 mixture of PanD and PanZ (final concentrations 15.8 and 31.7 μM respectively) in 50 mM Tris–HCl, 100 mM NaCl, 0.1 mM DTT, pH 7.4 with 50 μM AcCoA was incubated for 16 hours at 37 °C. The mixture was diluted into 50 mM K_2HPO_4, 100 mM NaCl, pH 7.4 to a final working concentration of 100 nM ADC and 200 nM PanZ (and consequently 0.32 μM AcCoA). Incubation samples were supplemented with AcCoA, PanZ and L-aspartate as indicated. ^{1}H NMR samples were made to 10% D_2O (630 μL reaction mixture with 70 μL D_2O) and the 1D ^{1}H NMR spectrum recorded immediately using a water suppression pulse sequence. Product formation was monitored by analysis of the ratio between the aspartate CαH and the β-alanine CH_2 signals determined by integration using Mestrenova (Mestrelab research software).

Emsley, P., Lohkamp, B., Scott, W.G., and Cowtan, K. (2010). Features and development of Coot. Acta Cryst. *D66*, 486-501.

Evans, P.R., and Murshudov, G.N. (2013). How good are my data and what is the resolution? Acta crystallographica. Section D, Biological crystallography *69*, 1204-1214.

Franke, D., and Svergun, D.I. (2009). DAMMIF, a program for rapid ab-initio shape determination in small-angle scattering. J. Appl. Cryst. *42*, 342-346.

Gasteiger, E., Hoogland, C., Gattiker, A., Duvaud, S.e., Wilkins, M., Appel, R., and Bairoch, A. (2005). Protein Identification and Analysis Tools on the ExPASy Server. In The Proteomics Protocols Handbook, J. Walker, ed. (Humana Press), pp. 571-607.

Houtman, J.C., Brown, P.H., Bowden, B., Yamaguchi, H., Appella, E., Samelson, L.E., and Schuck, P. (2007). Studying multisite binary and ternary protein interactions by global analysis of isothermal titration calorimetry data in SEDPHAT: application to adaptor protein complexes in cell signaling. Protein science : a publication of the Protein Society *16*, 30-42.

Keller, S., Vargas, C., Zhao, H., Piszczek, G., Brautigam, C.A., and Schuck, P. (2012). High-precision isothermal titration calorimetry with automated peak-shape analysis. Analytical chemistry *84*, 5066-5073.

Konarev, P.V., Volkov, V.V., Sokolova, A.V., Koch, M.H.J., and Svergun, D.I. (2003). PRIMUS: a Windows PC-based system for small-angle scattering data analysis. J. Appl. Cryst. *36*, 1277-1282.

Kozin, M.B., and Svergun, D.I. (2001). Automated matching of high- and low-resolution structural models. J. Appl. Cryst. *34*, 33-41.

Leslie, A.W., and Powell, H. (2007). Processing diffraction data with mosflm. In Evolving Methods for Macromolecular Crystallography, R. Read, and J. Sussman, eds. (Springer Netherlands), pp. 41-51.

Lovell, S.C., Davis, I.W., Arendall, W.B., 3rd, de Bakker, P.I., Word, J.M., Prisant, M.G., Richardson, J.S., and Richardson, D.C. (2003). Structure validation by Calpha geometry: phi,psi and Cbeta deviation. Proteins *50*, 437-450.

Monteiro, D.C., Rugen, M.D., Shepherd, D., Nozaki, S., Niki, H., and Webb, M.E. (2012). Formation of a heterooctameric complex between aspartate alpha-decarboxylase and its cognate activating factor, PanZ, is CoA-dependent. Biochem. Biophys. Res. Commun. *426*, 350-355.

Murshudov, G.N., Skubak, P., Lebedev, A.A., Pannu, N.S., Steiner, R.A., Nicholls, R.A., Winn, M.D., Long, F., and Vagin, A.A. (2011). REFMAC5 for the refinement of macromolecular crystal structures. Acta Cryst. *D67*, 355-367.

Nozaki, S., Webb, M.E., and Niki, H. (2012). An activator for pyruvoyl-dependent l-aspartate α-decarboxylase is conserved in a small group of the γ-proteobacteria including Escherichia coli. MicrobiologyOpen *1*, 298-310.

Petoukhov, M.V., Franke, D., Shkumatov, A.V., Tria, G., Kikhney, A.G., Gajda, M., Gorba, C., Mertens, H.D.T., Konarev, P.V., and Svergun, D.I. (2012). New developments in the ATSAS program package for small-angle scattering data analysis. J. Appl. Cryst. *45*, 342-350.

Saldanha, S.A., Birch, L.M., Webb, M.E., Nabbs, B.K., von Delft, F., Smith, A.G., and Abell, C. (2001). Identification of Tyr58 as the proton donor in the aspartate alpha-decarboxylase reaction. Chem. Commun. *18*, 1760-1761.
Schmitzberger, F., Kilkenny, M.L., Lobley, C.M., Webb, M.E., Matak-Vinkovic, D., Witty, M., Chirgadze, D.Y., Smith, A.G., Abell, C., and Blundell, T. (2003). Structural constraints on protein self-processing in L-aspartate-alpha-decarboxylase. EMBO J. *22*, 6193-6204.
Schneidman-Duhovny, D., Hammel, M., Tainer, J.A., and Sali, A. (2013). Accurate SAXS Profile Computation and its Assessment by Contrast Variation Experiments. Biophys. J. *105*, 962-974.
Studier, F.W. (2005). Protein production by auto-induction in high-density shaking cultures. Prot. Exp. Purif. *41*, 207-234.
Svergun, D. (1992). Determination of the regularization parameter in indirect-transform methods using perceptual criteria. J. Appl. Cryst. *25*, 495-503.
Vagin, A., and Teplyakov, A. (1997). MOLREP: an Automated Program for Molecular Replacement. J. Appl. Cryst. *30*, 1022-1025.
Volkov, V.V., and Svergun, D.I. (2003). Uniqueness of ab initio shape determination in small-angle scattering. J. Appl. Cryst. *36*, 860-864.
Webb, M.E., Yorke, B.A., Kershaw, T., Lovelock, S., Lobley, C.M.C., Kilkenny, M.L., Smith, A.G., Blundell, T.L., Pearson, A.R., and Abell, C. (2014). Threonine 57 is required for the post-translational activation of E. coli aspartate a-decarboxylase. Acta Cryst. *D70*, 1166-1172.
