# Supplementary material for: The Structure of the PanD/PanZ Protein Complex Reveals Negative Feedback Regulation of Pantothenate Biosynthesis by Coenzyme A
Source: Chem Biol. 2015 Apr 23;22(4):492–503. doi: 10.1016/j.chembiol.2015.03.017 (PMC4410942; doi:10.1016/j.chembiol.2015.03.017)
Supplement: Document S2. Article plus Supplemental Information [file mmc2.pdf]

Article

Chemistry & Biology

The Structure of the PanD/PanZ Protein Complex Reveals Negative Feedback Regulation of Pantothenate Biosynthesis by Coenzyme A

Graphical Abstract

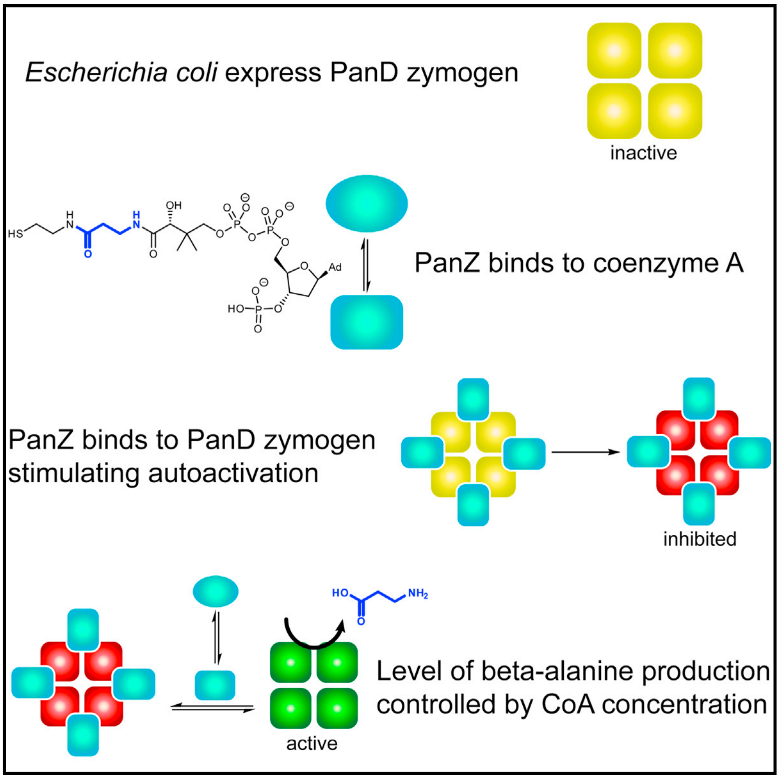

Authors

Diana C.F. Monteiro, Vijay Patel, ..., Arwen R. Pearson, Michael E. Webb

Correspondence

m.e.webb@leeds.ac.uk (M.E.W.),
arwen.pearson@cfel.de (A.R.P.)

In Brief

The structure of the complex of PanD and its activating factor PanZ is determined by Monteiro et al., revealing the basis for the CoA dependence of this interaction. This CoA-dependent interaction is shown to inhibit catalysis by the activated enzyme. This is the first report of regulation of pantothenate biosynthesis.

Highlights

- Structure of the PanD-PanZ.AcCoA complex is reported at a resolution of 1.6 Å
- Binding of AcCoA to PanZ is required to form the PanZ/PanD interface
- PanZ.AcCoA activates PanD via selection of a reactive conformation of PanD
- PanZ.AcCoA inhibits the activated enzyme, regulating pantothenate biosynthesis

Accession Numbers

4CRZ
4CS0

Monteiro et al., 2015, Chemistry & Biology 22, 492–503
April 23, 2015 ©2015 The Authors
http://dx.doi.org/10.1016/j.chembiol.2015.03.017

CellPress

Chemistry & Biology
Article

The Structure of the PanD/PanZ Protein Complex Reveals Negative Feedback Regulation of Pantothenate Biosynthesis by Coenzyme A

Diana C.F. Monteiro,[1,2,3] Vijay Patel,[1,3] Christopher P. Bartlett,[1,3] Shingo Nozaki,[5] Thomas D. Grant,[4] James A. Gowdy,[1,2,3] Gary S. Thompson,[1,3] Arnout P. Kalverda,[1,3] Edward H. Snell,[4] Hironori Niki,[5,6] Arwen R. Pearson,[1,3,7,*] and Michael E. Webb[1,2,*]
[1]Astbury Centre for Structural Molecular Biology, University of Leeds, Leeds LS2 9JT, UK
[2]School of Chemistry, University of Leeds, Leeds LS2 9JT, UK
[3]Faculty of Biological Sciences, University of Leeds, Leeds LS2 9JT, UK
[4]Hauptmann-Woodward Medical Research Institute, Buffalo, NY 14203, USA
[5]Microbial Genetics Laboratory, Genetic Strains Research Center, National Institute of Genetics, 1111 Yata, Mishima, Shizuoka 411-8540, Japan
[6]Department of Genetics, Graduate University for Advanced Studies (Sokendai), 1111 Yata, Mishima, Shizuoka 411-8540, Japan
[7]Present address: Hamburg Center for Ultrafast Imaging, Institute of Nanostructure and Solid State Physics, University of Hamburg, Luruper Chaussee 149, Hamburg 22761, Germany
*Correspondence: m.e.webb@leeds.ac.uk (M.E.W.), arwen.pearson@cfel.de (A.R.P.)
http://dx.doi.org/10.1016/j.chembiol.2015.03.017
This is an open access article under the CC BY license (http://creativecommons.org/licenses/by/4.0/).

SUMMARY

Coenzyme A (CoA) is an ubiquitous and essential cofactor, synthesized from the precursor pantothenate. Vitamin biosynthetic pathways are normally tightly regulated, including the pathway from pantothenate to CoA. However, no regulation of pantothenate biosynthesis has been identified. We have recently described an additional component in the pantothenate biosynthetic pathway, PanZ, which promotes the activation of the zymogen, PanD, to form aspartate α-decarboxylase (ADC) in a CoA-dependent manner. Here we report the structure of PanZ in complex with PanD, which reveals the structural basis for the CoA dependence of this interaction and activation. In addition, we show that PanZ acts as a CoA-dependent inhibitor of ADC catalysis. This inhibitory effect can effectively regulate the biosynthetic pathway to pantothenate, and thereby also regulate CoA biosynthesis. This represents a previously unobserved mode of metabolic regulation whereby a cofactor-utilizing protein negatively regulates the biosynthesis of the same cofactor.

INTRODUCTION

Coenzyme A (CoA) **1** is essential for the growth of all organisms and is derived from pantothenate **2** (Figure 1A). Pantothenate **2** is an essential nutrient for humans and is synthesized via a common pathway in plants, yeast, and bacteria (Webb et al., 2004). The majority of vitamin and amino acid biosynthetic pathways in bacteria are highly regulated either by metabolite-responsive transcription factors (Grose et al., 2005), product inhibition of key enzymes (Farrar et al., 2010), or metabolite-binding riboswitches (Winkler and Breaker, 2005). The pathway from pantothenate onward to CoA is regulated by feedback inhibition of pantothenate kinase (Rock et al., 2003; Yun et al., 2000) in all organisms, including bacteria, but no evidence for regulation of the pathway to pantothenate has been described.

In all organisms, the final step in pantothenate biosynthesis is the condensation of pantoate **3** and β-alanine **4** to form pantothenate **2**, but the source of β-alanine is different in bacteria, yeast, and plants. In bacteria, β-alanine is derived from L-aspartate **5** by the action of the enzyme aspartate α-decarboxylase (ADC) encoded by *panD* (Williamson and Brown, 1979). ADC is one of a small subset of enzymes containing a protein-derived, covalently linked pyruvoyl cofactor (van Poelje and Snell, 1990). This cofactor was first observed in histidine decarboxylase (Snell, 1986) and is present in a set of highly conserved enzymes, including *S*-adenosylmethionine decarboxylase (Pegg, 2009) and the membrane-bound phosphatidylserine decarboxylase (Schuiki and Daum, 2009). In all cases, the zymogens undergo an N→O acyl shift in their peptide backbones to form an ester (Figure 1B, **III**) that is then cleaved by elimination to generate the pyruvoyl cofactor (Figure 1B, **V**). The structural basis for this rearrangement in these enzymes has previously been investigated using a combination of site-directed mutagenesis (Gelfman et al., 1991; Schmitzberger et al., 2003; Webb et al., 2012, 2014), the structure of the zymogen (Schmitzberger et al., 2003), and the structure of inactive site-directed mutants (Schmitzberger et al., 2003; Tolbert et al., 2003a, 2003b; Webb et al., 2014). In some of these cases there is evidence for regulated activity. In the case of *S*-adenosylmethionine decarboxylase, both formation of the cofactor and subsequent catalytic activity are positively allosterically regulated in vitro by binding of the substrate for the succeeding step in the biosynthetic pathway (Bale et al., 2008; Stanley et al., 1994). In contrast, the catalytic activity of histidine decarboxylase is regulated by pH-dependent unfolding at neutral pH (Schelp et al., 2001). Similar peptide backbone modifications, leading to either autoproteolysis or backbone

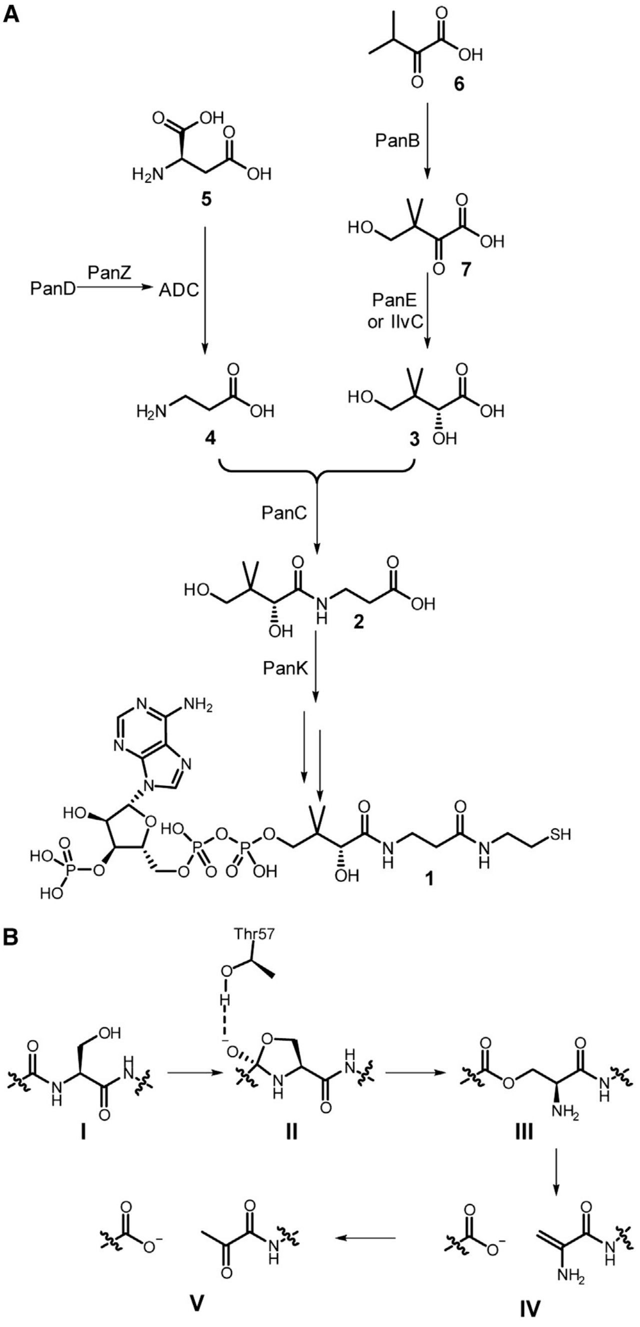

Figure 1. Biosynthetic Pathway to CoA and Mechanism for Cofactor Formation in PanD

(A) Biosynthetic pathway to pantothenate **2** and coenzyme A (CoA) **1** in bacteria. D-Pantoate **3** is generated from α-ketoisovalerate **6**, an intermediate in valine biosynthesis, via hydroxymethylation, to generate ketopantoate **7** and subsequent NADPH-dependent reduction. D-Pantoate is ligated to β-alanine **4**, which is generated via decarboxylation of L-aspartate **5** by aspartate α-decarboxylase (ADC). The principal characterized regulatory step is phosphorylation of pantothenate **2** to generate phosphopantothenate by pantothenate kinase (PanK), which is feedback-regulated by the terminal metabolite of the pathway CoA (Yun et al., 2000).

(B) Previously proposed mechanism of PanD activation. Thr57 hydrogen-bonds to Gly24, polarizing the carbonyl bond (Schmitzberger et al., 2003; Webb et al., 2014). Ser25 attacks Gly24 to a ring intermediate **II** which opens to the ester intermediate **III** (Albert et al., 1998). This is cleaved by elimination to a dehydroalanine **IV**, which can then hydrolyze to the pyruvoyl cofactor **V**.

rearrangement, have been observed in a diverse range of systems; including the post-translational processing of inteins (Paulus, 2000), post-translational activation of the N-terminal nucleophile hydrolases (Buller et al., 2012; Kim et al., 2006), recA-mediated cleavage of the DNA-binding protein umuD (McDonald et al., 1998), and autoproteolysis of SEA domains (Johannson et al., 2009; Levitin et al., 2005). In most cases these post-translational modifications are autocatalytic, but the reaction rates of a subset of enzymes have been shown to be enhanced by accessory proteins (McDonald et al., 1998), although the structural basis for such enhancement has not been determined.

Since the first identification of pyruvoyl-dependent enzymes (van Poelje and Snell, 1990; Williamson and Brown, 1979), it has been widely presumed that their post-translational rearrangement is also autocatalytic (Ramjee et al., 1997). Recently, however, accessory proteins essential for the activation of some members of this class of proteins have been identified (Nozaki et al., 2012; Stuecker et al., 2012a; Trip et al., 2011). HdcB, the essential accessory protein for histidine decarboxylase activation (HdcA) from *Streptococcus thermophilus*, was identified by Trip et al. (2011). Subsequently, the accessory protein for maturation of PanD, PanZ, was described independently in *Escherichia coli* (Nozaki et al., 2012) and the closely related *Salmonella typhi* (Stuecker et al., 2012a). PanZ is essential for activation of the zymogen PanD to form ADC in vivo, and its deletion leads to β-alanine auxotrophy (Nozaki et al., 2012). In this article, we report the structure of the protein complex formed between PanZ and PanD, which has allowed us to determine both the basis for CoA-dependent interaction and how the tight interaction of PanD and PanZ leads to activation of PanD to form ADC. Secondly, we report our investigation of the role of PanZ in global regulation of the pantothenate, and thereby the CoA, biosynthetic pathway, leading us to propose a novel mode of metabolic regulation via ligand-dependent protein-protein interaction.

RESULTS

Architecture of the PanD-PanZ Complex Reveals Origin of CoA-Dependent Interaction

PanD (encoded by *panD*) is post-translationally modified to form its catalytically active form, ADC, by cleavage of the peptide backbone between residues Gly24 and Ser25, leading to the formation of a pyruvoyl cofactor from Ser25 (Figure 1B). The residues in PanD required for activation have been previously explored by mutagenesis. As expected, mutation of Ser25 to alanine leads to loss of activation (Schmitzberger et al., 2003) but the only proximal residue found to be required for activation is Thr57, where mutation to the isosteric valine leads to complete loss of activation (Webb et al., 2014). We have previously used this inactivatable PanD(T57V) mutant to characterize the interaction of PanD with PanZ (Monteiro et al., 2012), and demonstrated that interaction is dependent on the presence of CoA. We observed substoichiometric binding between ADC and PanZ, probably due to the formation of disulfide-linked CoA dimers in solution. As PanZ binds acetyl CoA (AcCoA) with equal affinity

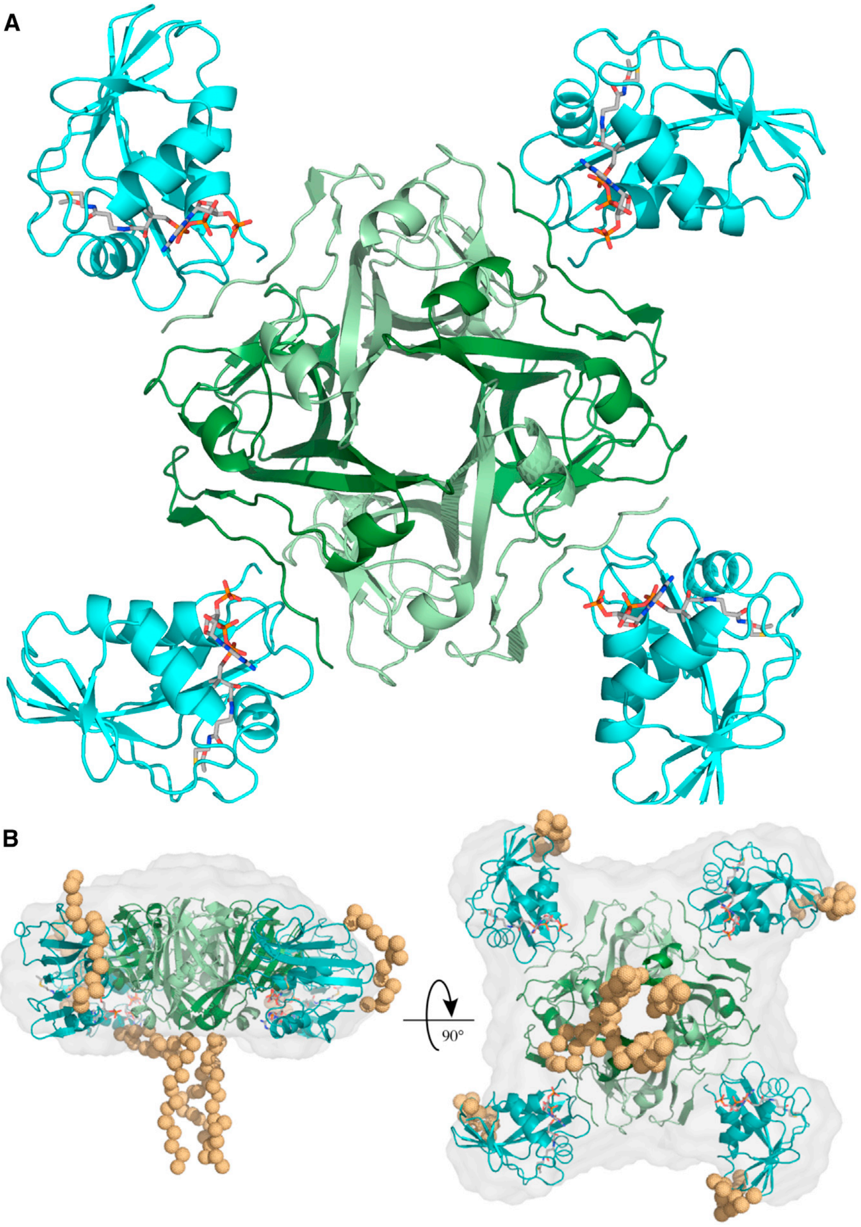

Figure 2. Architecture of the PanD-PanZ Protein Complex

(A) Overview of the protein complex showing the four PanD protomers and highlighting the proximity of the PanD protomer-protomer interface to the PanD-PanZ interface. The PanD tetramer binds four PanZ proteins and the interaction is promoted by AcCoA molecules. In all figures, PanD(T57V) and PanZ are shown in green and blue, respectively, and AcCoA as sticks with carbons colored gray.

(B) Side and top views of the protein complex showing the modeled crystallographically unresolved affinity tags as orange spheres and the calculated SAXS envelope, with a final χ value of 1.76 (see also Figures S1 and S2).

to CoA (Monteiro et al., 2012), we re-investigated the interaction of the proteins in the presence of AcCoA instead, using both the previously described PanD(T57V) mutant and the also inactivatable PanD(S25A) mutant. For both proteins, global fitting with a 1:1 binding model (Houtman et al., 2007) yielded robust estimates for dissociation constants of 35 ± 4 nM for the PanD(T57V)-PanZ.AcCoA interaction and 157 ± 5 nM for the PanD(S25A)-PanZ.AcCoA interaction (Figure S1).

Following this, we characterized the structural basis of protein complex formation. Bipyrimidal crystals were reproducibly obtained using a 1.1-fold excess of PanZ over PanD(T57V) and a 2-fold excess of AcCoA, with respect to PanZ. X-Ray diffraction data were collected from these crystals to 1.7 Å resolution at room temperature using an in-house X-ray source. The crystals contained a single PanD protomer and one PanZ molecule per asymmetric unit (Table S1). The structure of the protein complex revealed a highly symmetric heterooctameric complex. The four active sites of the PanD heterotetramer and the loops that are cleaved to generate them lie at the interface of each pair of PanD protomers. The PanD-PanZ protein complex is a cross-shaped heterooctamer, with one PanZ molecule bound to each of the PanD protomer-protomer interfaces (Figure 2A).

We used small-angle X-ray scattering (SAXS) to confirm that the architecture observed in the crystal corresponds to that of the complex in solution (Figure 2B). A solution sample of the two proteins at a 1:1 ratio in the presence of two equivalents of AcCoA was used for data collection at varying concentrations. The calculated atomic scattering factors for the crystallographic model fitted well to the observed scattering at low protein concentrations, with small deviations at high and low scattering angles (Figure S2). These deviations could be accounted for by inclusion of both the crystallographically unresolved affinity purification tags (shown modeled in Figure 2B) and a small population of a dimer of the PanD-PanZ complex (two copies of the heterooctamer), consistent with the packing observed in the crystal structure. The population of this dimer species increased in scattering data recorded at higher concentrations, suggesting a concentration-dependent aggregation phenomenon.

Nuclear Magnetic Resonance Demonstrates that CoA Is an Absolute Requirement for Complex Formation

As expected from our preliminary biophysical characterization (Monteiro et al., 2012), each molecule of PanZ binds one molecule of AcCoA. The binding site for AcCoA is very close to the protein-protein interface, accounting for the previously observed CoA dependence of the interaction (Monteiro et al., 2012). The structure of PanZ, when in complex with PanD(T57V), is largely

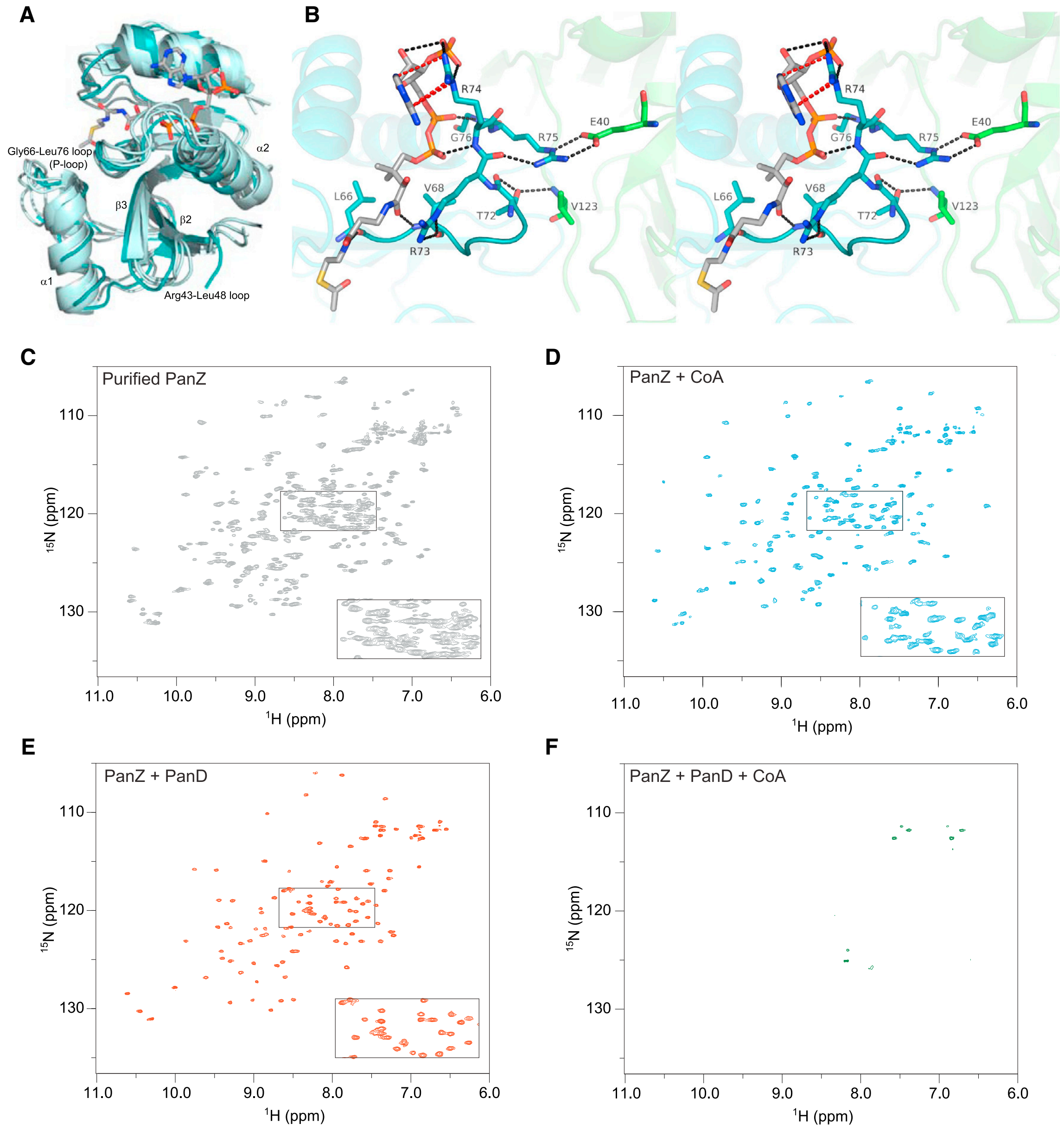

Figure 3. NMR Analysis of the PanD-PanZ.AcCoA Complex

(A) Overlay of the crystal structure of PanZ in complex with PanD with the NMR solution structure of the PanZ.AcCoA complex (PDB code 1K5T).

(B) Stereoscopic view of the CoA-binding site of PanZ showing key hydrogen-bonding interactions. Binding of AcCoA is dependent upon hydrogen-bonding interactions to the PanZ Leu66-Gly76 loop, which also forms hydrogen bonds with PanD. The acetyl group of AcCoA is distal to the PanZ-PanD interface. AcCoA, PanD, and PanZ are colored gray, green, and cyan, respectively.

(C) ^{1}H-^{15}N HSQC spectrum of PanZ after purification from *E. coli* shows a complex spectrum due to the presence of co-purified CoA derivatives (Monteiro et al., 2012), leading to a mixture of free PanZ together with PanZ.RCoA.

(D) ^{1}H-^{15}N HSQC spectrum after addition of CoA is simplified to yield a well-dispersed spectrum of the PanZ.RCoA complex.

(E) Addition of excess PanD to PanZ yields a well-dispersed ^{1}H-^{15}N HSQC spectrum of apo-PanZ, which is distinct from that observed after addition of CoA. PanD-PanZ.AcCoA is not observed.

(F) Addition of both excess CoA and PanD to PanZ leads to loss of all signals in the ^{1}H-^{15}N HSQC due to formation of the PanD-PanZ.AcCoA complex (~124 kDa) in which presumed fast relaxation leads to signal broadening and consequent loss of signal (see also Figure S3).

isostructural with the monomeric structure for PanZ previously elucidated by nuclear magnetic resonance (NMR) in the presence of CoA (PDB 2K5T, Figure 3A), suggesting that binding of CoA causes PanZ to adopt an ADC-binding conformation. In particular, binding of AcCoA appears to stabilize the PanZ Leu66-Gly76 loop, which forms contacts with PanD (Figure 3B and Figure S3). At the same time, the acetyl portion of the cofactor is held away from PanD on the distal face of PanZ, suggesting that acetylation is not required for activation, consistent with the findings of Stuecker et al. (2012b).

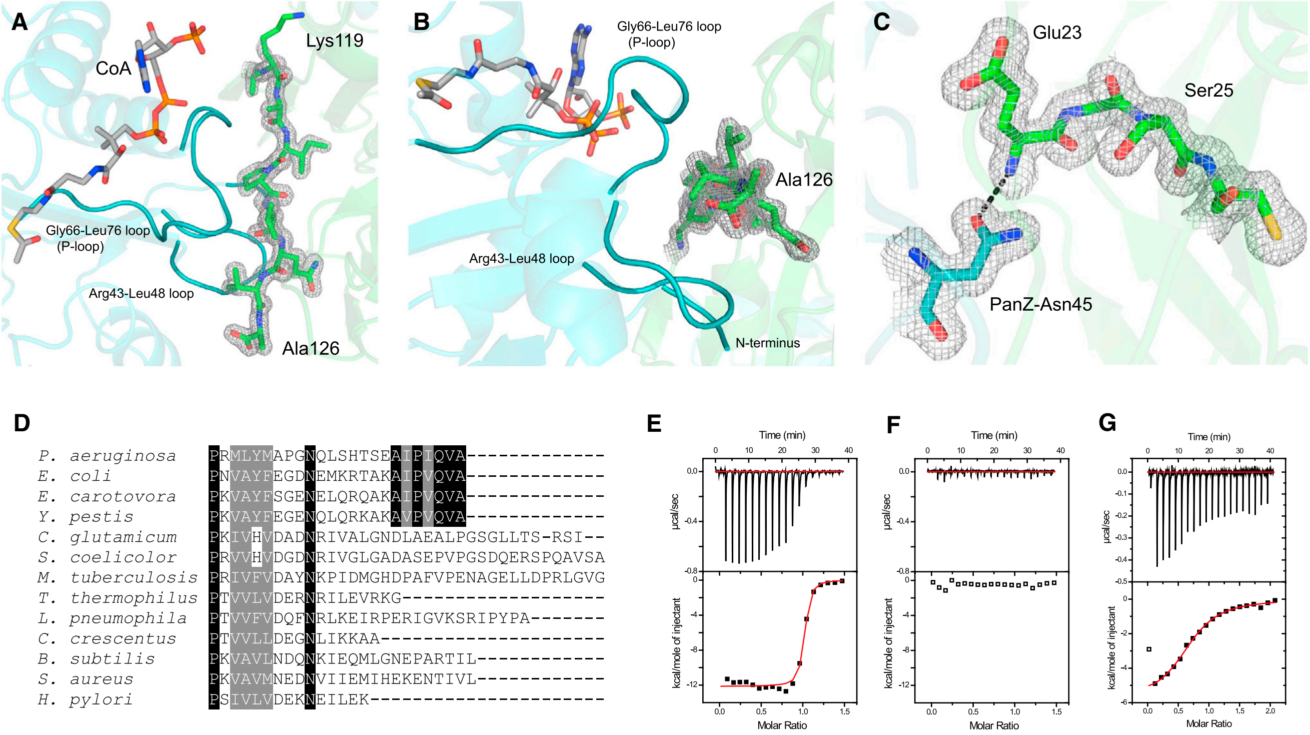

Figure 4. Detailed Analysis of the PanD-PanZ Interaction by Mutagenesis

(A and B) Detail of the interface between PanD and PanZ. AcCoA, PanD, and PanZ are colored gray, green, and cyan, respectively. The C terminus of PanD (Lys119-Ala126, shown as sticks, and $2F_0$-F_c electron density maps contoured at 1 root-mean-square deviation [rmsd]), is ordered and sandwiched between the PanZ Leu66-Gly76 and Arg43-Leu58 loops.

(C) PanZ-Asn45 has been identified as being critical for activation (Nozaki et al., 2012). This residue forms a hydrogen bond with the main-chain amide of PanD-Glu23 adjacent to the position of chain cleavage between PanD-Gly24 and Ser25. PanZ-Asn45 and PanD residues 23–26 are shown as sticks with $2F_0$-F_c electron density maps contoured at 1 rmsd.

(D) Sequence alignment of the C-terminal portion of representative PanD orthologs. The C-terminal seven amino acids are fully conserved in those organisms which encode PanZ (*Pseudomonas*, *Erwinia*, *Salmonella*, *Escherichia*, *Yersinia* and related Enterobacteriaceae).

(E) ITC isotherm for titration of 263 μM PanZ into 35 μM PanD-T57V in the presence of excess AcCoA fitted with a single-site binding model indicates an interaction with 35 nM affinity.

(F) ITC isotherm of titration of 263 μM PanZ into 35 μM PanD-T57V/K119Stop. Deletion of residues K119 to Ala126 leads to loss of interaction.

(G) Titration of 400 μM PanZ-N45A into 40 μM PanD-T57V reveals a decreased affinity of 4.4 ± 0.4 μM. Mutation of PanZ-Asn45 leads to loss of high-affinity interaction.

While we crystallized the complex of PanZ and PanD with bound AcCoA and observed CoA concentration effects on the interaction by isothermal titration calorimetry (ITC) (Monteiro et al., 2012), this does not demonstrate unambiguously that the proteins cannot interact in the absence of CoA. We therefore generated ^{15}N-labeled PanZ and used a ^{1}H-^{15}N heteronuclear single quantum coherence (HSQC) experiment to assess the homogeneity of the protein preparation. As previously reported by Cort et al. (2009), we observed more peaks than expected for the size of the protein (Figure 3C), presumably due to the presence of both CoA-bound- and *apo*-PanZ in the sample. When PanZ is overexpressed and purified, it is isolated with a substoichiometric quantity of what are presumed to be a mixture of CoA and its thioesters (annotated as RCoA), as shown by our previous observation of substoichiometric binding of AcCoA and CoA to the purified protein (Monteiro et al., 2012). Addition of excess AcCoA led to simplification of the spectrum (Figure 3D) due to formation of a single species in the sample tube. Finally, we added excess PanD(T57V) to AcCoA-saturated PanZ. This led to loss of almost all signals from the NMR spectrum (Figure 3F): the PanD-PanZ.AcCoA complex is approximately 124 kDa, and the slow tumbling rate of this species in solution leads to extensive broadening of the signals such that they are no longer observed.

This loss of signal upon formation of the complex provides an effective assay for complex formation. If AcCoA is not essential for PanD-PanZ complex formation, addition of PanD(T57V) to the mixed population of PanZ and PanZ.RCoA obtained upon protein purification should lead to loss of all signals. Addition of PanD(T57V) to the mixed population of ^{15}N-labeled PanZ also led to simplification of the spectrum (Figure 3E), similar to that observed by addition of AcCoA; however, a different set of peaks were resolved in the spectrum, presumably corresponding to apo-PanZ. To confirm that this simplification was due to insufficient CoA rather than substoichiometric PanD(T57V), we then added an excess of AcCoA. Once again, this led to loss of the observed spectrum (not shown), demonstrating unambiguously that CoA or a derivative is required for formation of the PanD-PanZ complex.

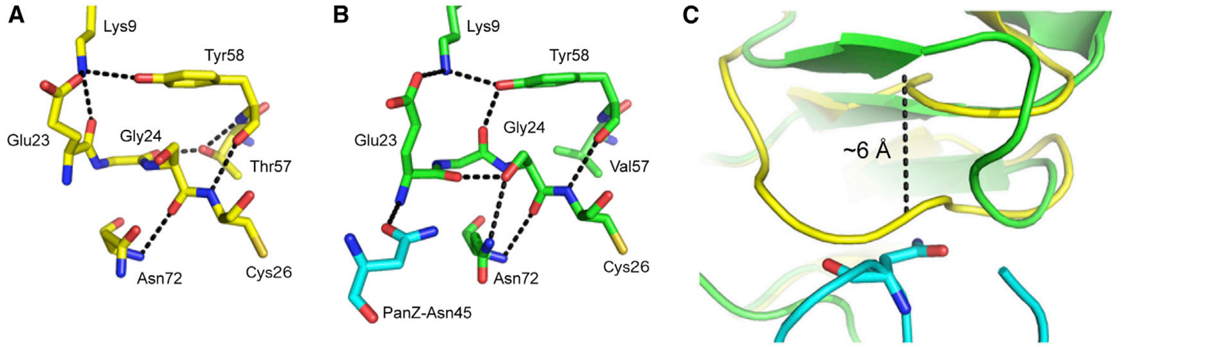

Figure 5. The Binding of PanZ Promotes PanD Processing: Comparison with Previously Determined Structures

(A) The PanD processing loop (residues Glu23 to Cys26) from the crystal structure of the wild-type zymogen determined by Schmitzberger et al. (2003).

(B) The PanD processing loop (residues Glu23 to Cys26) from the PanD(T57V)-PanZ protein complex (green). PanZ-Asn45 (cyan) forms a single hydrogen bond to the PanD-Glu23 backbone (see Figure 4C). The processing loop is compacted relative to the wild-type structure and the carbonyl of Gly24 now interacts with the side chain of Tyr58 rather than Thr57.

(C) Comparison between PanD(T57V)-PanZ (green) and the high-occupancy conformation adopted by the zymogen (1PPY, yellow). The non-processing-prone conformation is incompatible with the binding of PanZ (cyan), sterically clashing with the PanZ-Asn45 loop. In the activatable conformation, the loop moves upward to form a new β sheet, 6.4 Å away from its original position (see also Figures S4–S6).

Structural Basis for Activation of ADC by PanZ

The PanD-PanZ interaction is mediated by the PanZ N terminus, Arg43-Leu48 loop, and Gly66-Leu76 loop (which also interacts with AcCoA). Together, these elements interact with the surface of PanD to either side of the PanD C-terminal tail (residues Lys119 to Ala126, Figures 4A and 4B). This portion of ADC is normally completely unstructured and has only previously been observed in the structure of the ADC(N72A) mutant, in a different, extended conformation that was stabilized by crystal packing contacts (Webb et al., 2012). The C-terminal sequence of PanD is hydrophobic (^{119}KAIPVQVA^{126}) and is fully conserved among organisms that encode PanZ (Figure 4D) (Nozaki et al., 2012), despite lying outside what can be regarded as the core fold of the protein. The PanD C-terminal region is at the core of the protein-protein interaction and becomes completely buried as a result of complex formation. We used calorimetry to investigate the importance of the C terminus of PanD for complex formation. We generated the site-directed mutant PanD(T57V/K119Stop) in which the seven C-terminal amino acids are deleted, and repeated the titration of PanZ.AcCoA into the purified PanD(T57V/K119Stop). No interaction between the proteins could be observed, suggesting that the C terminus of PanD is essential for binding (Figure 4F).

The remainder of the protein-protein interface is maintained by numerous hydrogen-bonding interactions between conserved and semi-conserved residues in PanD and PanZ. One key hydrogen-bonding interaction is between the side chain of PanZ-Asn45 and the backbone amide proton of PanD-Glu23 (Figure 4C). Both residues are conserved in organisms that express PanZ. Mutation of PanZ-Asn45 has previously been shown to result in cells unable to generate active ADC, suggesting that this contact might be key to the activation of PanD (Nozaki et al., 2012). To test this hypothesis, we investigated the interaction of a PanZ(N45A) mutant with PanD(T57V) using ITC (Figure 4G). This revealed an 80-fold decrease in affinity, to ~4 μM. The most recent consensus measurements of protein abundance derived from proteomic studies (Wang et al., 2012) for PanD and PanZ are 240 and 72 ppm, respectively, i.e. concentrations of approximately 500 and 150 nM. The lower affinity of the mutant PanZ(N45A) for PanD is therefore sufficient to prevent formation of the complex, and explains the inability of PanZ(N45A) to effectively complement the β-alanine auxotrophy of Δ*panZ* strains (Nozaki et al., 2012).

Previous studies of the ADC activation mechanism have focused on the identification of residues that are required to catalyze the rearrangement of the peptide chain. It was originally hypothesized that at least two catalytic residues would be necessary for general acid-base catalyzed ester formation (Schmitzberger et al., 2003; Webb et al., 2012, 2014). However, only mutagenesis of Thr57 leads to loss of activation. To confirm that the observed PanD(T57V) conformation when in complex with PanZ is solely caused by the protein-protein interaction rather than as a result of the site-directed mutation, we determined the structure of a second non-activatable mutant, PanD(S25A), in complex with PanZ.AcCoA. Diffraction data were collected at room temperature in-house to 2.1-Å resolution. The overall architecture and major protein-protein interactions were completely consistent between the two complexes (PanD(S25A)-PanZ and PanD(T57V)-PanZ). However, owing to the change in the available hydrogen-bonding interactions, slightly different conformations of the active-site loop were seen (Figure S4). Nevertheless, in both structures one of the two residues at the site of cleavage adopted a Ramachandran-disallowed conformation (Ramachandran et al., 1963) (Figure S5), suggesting that the PanD activation region is forced into an unfavored and, therefore, high-energy conformation upon binding of PanZ. This destabilization may lower the energy barrier for activation of PanD to ADC.

To further investigate how the activation reaction is catalyzed, we compared the structure of the PanD(T57V)-AcCoA.PanZ complex with that of the previously reported structure of the wild-type PanD zymogen in the absence of PanZ (Schmitzberger et al., 2003). The location of the peptide backbone in the immediate region of eventual peptide cleavage (Glu23-Cys26) in the PanD zymogen is largely similar to that observed in the PanD-AcCoA.PanZ complex, although the exact conformations of individual residues, particularly the carbonyl of Gly24 (Figures 5A and 5B), have changed. In contrast, the position of the

CellPress

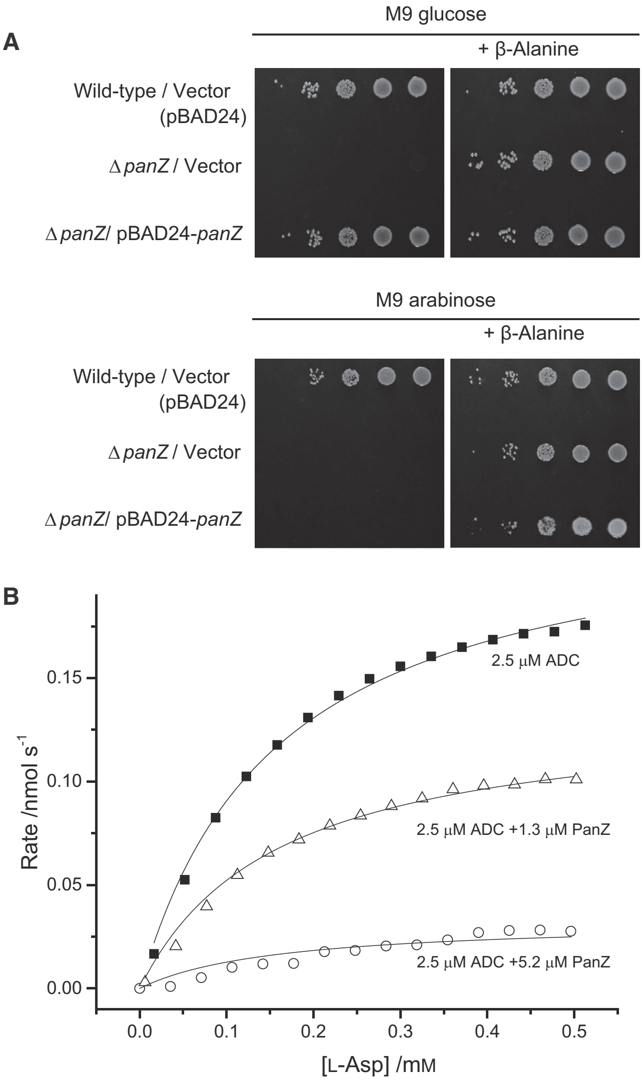

Figure 6. PanZ Inhibits Catalytic Activity by Activated PanD

(A) Overexpression of PanZ in *E. coli* leads to inhibition of cellular growth. Leaky expression of PanZ under the arabinose promoter on M9 glucose media leads to complementation of β-alanine auxotrophy (top panel); induction of protein overexpression via growth on M9 arabinose media (bottom panel) leads to maintenance of the β-alanine auxotrophy. Overnight cell culture in L broth was spotted at a series of 1:10 dilutions.

(B) Effect of PanZ.AcCoA on catalysis by activated ADC using an ITC-based assay. Addition of PanZ.AcCoA to ADC leads to inhibition of catalytic formation of β-alanine. Data are derived from the steady-state heat production following sequential addition of fixed volumes of L-aspartate to the enzyme in the instrument. Individual curves are fitted to the Michaelis-Menten curve with a shared value of K_M (see also Figure S7).

PanD loop between Thr16 and Tyr22 is wholly different. In the published PanD zymogen structure, this region adopts two distinct conformations (Schmitzberger et al., 2003). One is well resolved and consists of a loop without defined secondary structure (Figure S6A), whereas the second, low-occupancy, conformation is poorly defined in the electron density (Figure S6B). This second low-occupancy conformation corresponds to the conformation observed, at full occupancy, in the structures of fully activated ADC (Albert et al., 1998) and our newly determined structures of the PanD-PanZ.AcCoA complex. The N terminus of PanZ and its Arg43-Leu48 loop are positioned in the space that would be occupied by the high-occupancy conformation of the uncleaved peptide chain of free PanD (Figure 5C). We hypothesize that, in the absence of PanZ, this portion of inactivated PanD can explore a large number of alternative conformations. Binding of PanZ results in restriction of the PanD Thr16-Tyr22 loop to conformations that resemble the activated form. This, in turn, places the Glu23-Ser25 backbone into a conformation that favors the activation reaction. In the two uncleavable PanD site-directed mutants used in this study, the unfavorable nature of this adopted conformation is reflected by the formally disallowed peptide backbone angles observed for either PanD-Gly24 or Ser25. Since the formation of the PanD-PanZ complex depends on the presence of CoA, this suggests that activation of PanD will in turn be controlled by the intracellular CoA concentration.

Inhibition of ADC Catalysis Reveals a Second Global Role for PanZ in Regulation of Pantothenate Biosynthesis

The CoA dependence of the PanD-PanZ interaction and, therefore, PanD activation suggests the presence of a positive feedback mechanism in pantothenate biosynthesis. To further investigate the cellular function of PanZ, we first re-examined the effect of PanZ overexpression in vivo. Deletion of *panZ* leads to cells auxotrophic for β-alanine, and we have previously shown that leaky, uninduced expression of PanZ is sufficient to complement the β-alanine auxotrophy of *E. coli* Δ*panZ* cells (Nozaki et al., 2012). In this case, weak expression of His-tagged PanZ under the control of the arabinose promoter in glucose minimal media leads to complementation of the *panZ*$^-$ phenotype (Figure 6A). However, when PanZ is overexpressed following growth on arabinose, complementation is not observed and, in fact, overexpression of this essential protein leads to retention of β-alanine auxotrophy. Since the conformation of the inactivated zymogen in the PanD-PanZ.AcCoA complex is similar to that of the free activated protein, we hypothesized that PanZ.AcCoA might also interact with activated ADC to regulate catalysis.

We investigated the effect of PanZ.AcCoA on catalysis of L-aspartate decarboxylation by ADC. This activity can be characterized using calorimetry to directly measure the enthalpy of decarboxylation and protonation (Todd and Gomez, 2001), yielding kinetic parameters similar to those obtained by stopped assays of product formation. Addition of 0.5 equivalents of PanZ.AcCoA to fully activated ADC led to ~50% reduction in the maximal ADC activity, whereas addition of 1 or 2 equivalents of PanZ.AcCoA led to a further drop in ADC activity to ~5%–10% maximal activity (Figure 6B). ADC is a relatively inefficient enzyme (k_{cat} ~ 0.2 s^{-1}) (Ramjee et al., 1997; Williamson and Brown, 1979), and as a result the enzyme concentration required for signal detection in the ITC assay is high (2.5 μM) relative to the affinity of PanZ.AcCoA for PanD (~100 nM). We therefore used ^{1}H NMR to characterize the effect of PanZ.AcCoA on ADC activity at lower enzyme concentrations (100 nM ADC). Using this assay we observed inhibition of ADC-catalyzed turnover of L-aspartate by PanZ at low L-aspartate concentration (500 μM,

$3K_M$) but not at high L-aspartate concentration (10 mM, $60K_M$), suggesting a competitive mode of inhibition with respect to L-aspartate (Figure S7).

DISCUSSION

The structure of the PanD-PanZ.AcCoA complex resolves two major questions in pantothenate biosynthesis: how does a CoA-dependent protein-protein interaction promote activation of PanD to form catalytically active ADC, and how is β-alanine biosynthesis regulated in enteric bacteria? We have identified an additional level of regulation mediated by this small-molecule-dependent protein-protein interaction, and this leads us to propose the first model for global regulation of this biosynthetic pathway.

How and Why Does CoA Binding Mediate Protein-Protein Interaction?

PanZ is a member of the GNAT superfamily of acetyl transferases (Vetting et al., 2005). It retains many of the conserved active-site residues of this superfamily, but Stuecker et al. (2012b) have previously shown, in the *Salmonella typhimurium* system, that mutation of potential substrate lysine residues in PanD does not affect the PanD activation reaction and that acetylation of PanD is not required for activation. In accordance with this observation, in the PanD-PanZ.AcCoA complex the acetyl group of AcCoA is sited away from the PanD-PanZ interface. The proximity of CoA to the PanD-PanZ interface and the similarity between the structure of PanZ in the PanD-PanZ.AcCoA complex and that of the PanZ.CoA complex previously determined by NMR suggest that binding of either AcCoA or CoA places PanZ in a PanD binding-competent conformation. Major changes are observed in the ^{1}H-^{15}N HSQC spectrum of PanZ upon binding CoA, corresponding to a substantial conformational change. Such a change would be consistent with data from other members of the GNAT family for which the structure of both apoenzyme and binary complexes have been determined (Vetting et al., 2005). In many of these cases, binding of AcCoA or CoA leads to substantial rearrangements in the P loop (which binds the pyrophosphate moiety of CoA). In the case of the PanD-PanZ.AcCoA complex, this loop forms key interactions with the C terminus of PanD, suggesting that CoA mediates this protein-protein interaction by structuring this loop.

How Does Interaction of PanZ with PanD Promote Activation of PanD to Form Catalytically Active ADC?

Direct interaction of PanD with the PanZ Arg43-Leu46 loop promotes PanD to adopt a reactive conformation, which leads to activation. In the absence of PanZ.AcCoA, this conformation is still accessible to PanD and is observed as a minor population in the structure of the uncleaved zymogen (Schmitzberger et al., 2003) in comparison with the major, unrestrained conformation (Figure S6B). This conformation places the PanD Glu23-Cys26 loop in the correct spatial arrangement for post-translational cleavage, and destabilizes the PanD Gly24-Ser25 peptide bond for facilitated nucleophilic attack and subsequent cleavage.

Observation of multiple zymogen backbone conformations is consistent with other autoprocessing systems. The active-site peptides of N-terminal nucleophile hydrolases have been shown to explore multiple conformations, only one of which is competent for autoproteolysis (Buller et al., 2012). In the case of PanD, sampling of this conformation probably accounts for the observed slow thermal activation to ADC after purification in vitro (Ramjee et al., 1997). The rate of thermal activation is, however, insufficient to support pantothenate biosynthesis in vivo in the absence of PanZ (Nozaki et al., 2012). Nonetheless, PanD from other organisms can autoactivate, suggesting that subtle differences in the side chain interactions within the PanD activation loop may induce formation of the mature-enzyme-like β sheet between Thr16-Asp19 and Ile69-Asn72, leading to autocatalytic activation.

The structure of the PanD-PanZ.AcCoA complex reported here also allows us to propose a new mechanism for the activation reaction (Figure 7A). In the structure of the zymogen (PDB 1PPY), the carbonyl of Gly24 forms a hydrogen bond with the side chain of Thr57, which was previously proposed to polarize the carbonyl of Gly24 to favor nucleophilic attack by the hydroxyl of Ser25 (Figures 7A **I** and 7B) (Schmitzberger et al., 2003; Webb et al., 2014). However, the Ser25 hydroxyl is poorly positioned in the zymogen structure. It is 4.3 Å away from the Gly24 carbonyl carbon and in the plane of the carbonyl bond approximately 4 Å from where significant orbital overlap leading to reaction could occur. Substantial further backbone rearrangement would be required for the N→O acyl shift to occur. In contrast, in the PanD-PanZ.AcCoA structure the hydrogen bond from Gly24 to Thr57 is no longer present, due to a 120° rotation of the Gly24 carbonyl (Figures 7A **II** and 7C). Instead, a new hydrogen bond is formed between the Gly24 carbonyl and Tyr58. The Gly24 rotation places the Ser25 hydroxyl 3 Å from the Gly24 carbonyl carbon, and only approximately 1.5 Å from a position with significant orbital overlap between the Ser25 lone pair sp^3-orbital and the carbonyl π^* orbital. Ser25 is also hydrogen bonded to the carbonyl of Glu23, which may act as a proton-shuttling residue to transfer the proton from Ser25 during the rearrangement reaction.

What, then, is the role of Thr57 in the first stage of the activation reaction? There is no evidence for ester formation in the structure of PanD(T57V), either alone (Webb et al., 2014) or in complex with PanZ.AcCoA. Furthermore, incubation of the PanD(T57V)-PanZ.AcCoA complex with hydroxylamine does not lead to chemically induced cleavage of PanD (data not shown), demonstrating that an ester intermediate is not formed. If Thr57 is not required to polarize the carbonyl of Gly24 toward nucleophilic attack, it must function in the next step of the reaction and deliver a proton to the amide nitrogen as the intermediate oxyoxazolidine ring (Figure 7A **III**) is opened. The pK_a of the amide anion formed is such that it could readily deprotonate a threonine side chain. The resulting deprotonated Thr57 residue (Figure 7A **IV**) could then act immediately to catalyze elimination of the ester to generate a dehydroalanine residue (Figure 7A **V**), which subsequently hydrolyzes non-enzymatically to generate the pyruvoyl group. While unusual, this mechanism effectively couples the ester formation and elimination steps, avoiding the formation of the hydrolyzed products observed from thermal cleavage (Ramjee et al., 1997).

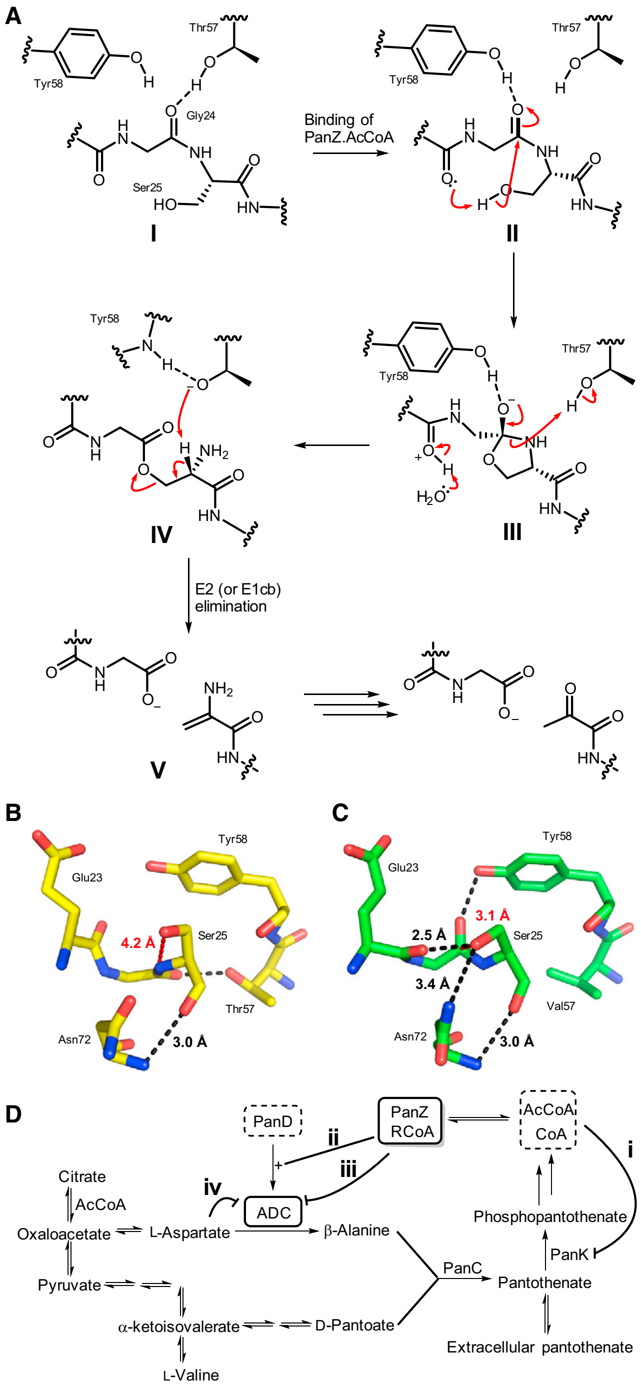

Figure 7. Proposed Role for PanZ in Activation of PanD and Regulation of Pantothenate Biosynthesis

(A) Revised model for activation of aspartate α-decarboxylase. Before binding of PanZ, the carbonyl of Gly24 forms a hydrogen bond to the side chain of Thr57 (state **I**, see B). Binding of PanZ induces a conformation change in the peptide chain rotating the carbonyl of Gly24 to hydrogen-bond to Tyr58 and shifting the hydroxyl of Ser25 to a position where reaction is possible (state **II**, see C). Following attack of the Ser25 hydroxyl on the carbonyl of Gly24 to form the oxyoxazolidine intermediate **III**, the side chain of Thr57 donates a proton to facilitate cleavage of the C-N bond to form the ester intermediate **IV**. The deprotonated Thr57 residue is then able to remove the α proton from Ser25 to cleave the peptide chain and generate a dehydroalanine residue **V**, which hydrolyzes to form the active enzyme.

(B) Structure of the PanD activation loop prior to binding of PanZ showing key hydrogen-bonding interactions. The nucleophilic Ser25 hydroxyl is 4.2 Å from the carbonyl of Gly24 and in the plane of the bond.

(C) Structure of the PanD activation loop following binding of PanZ. Binding of PanZ leads to formation of a hydrogen bond between the carbonyl of Glu23 and the side chain of Ser25. Peptide backbone reorientation places the Ser25 nucleophilic group 3 Å from the Gly24 carbonyl carbon and above the plane of the bond in a position where minimal conformational change is required for reaction.

(D) Proposed model for global regulation of pantothenate and CoA biosynthesis in *E. coli* by PanZ. (i) The pathway from pantothenate to CoA is controlled by feedback regulation of pantothenate kinase (Rock et al., 2003). PanZ controls both the activation (ii) and catalytic activity (iii) of PanD in a CoA-dependent fashion. In contrast to β-alanine, formation of D-pantoate is reversible and β-alanine is the sole committed step in the biosynthetic pathway. CoA-dependent regulation by PanZ can therefore control the flux through the whole pantothenate biosynthetic pathway. Finally, the active form of PanD, ADC, undergoes substrate-mediated inhibition (iv) after approximately 300 turnovers, limiting the total flux through the pathway (Konst et al., 2009).

How Does Interaction of PanZ and PanD Regulate the Pantothenate Biosynthetic Pathway?

The biosynthesis of CoA has previously been shown to be regulated at the level of pantothenate phosphorylation to form phosphopantothenate (Rock et al., 2003; Yun et al., 2000). Pantothenate kinase catalyzes this step and is allosterically regulated by CoA. Until *panZ* was identified there were no known regulatory mechanisms for the steps upstream of pantothenate. The pantothenate biosynthetic pathway is relatively simple (Figures 1 and 7D). α-Ketoisovalerate, the oxoacid of valine, is hydroxymethylated and reduced to form pantoate, which is condensed with β-alanine to form pantothenate. The two reactions that form pantoate are reversible and so, in the absence of β-alanine, these metabolites exist in equilibrium with those in the pathway to valine (Figure 7D). Since the cellular pools of L-aspartate and D-pantoate are in equilibrium with other primary metabolites, formation of β-alanine is the only committed step in the pathway. How, then, can PanZ regulate this step? We have characterized two distinct functions for PanZ: activation of PanD to form ADC and subsequent inhibition of ADC. We therefore propose a new regulatory model whereby ADC activity is limited and regulated by the concentration of CoA in the cell (Figure 7D).

We hypothesize that such inhibitory activity is actually the primary metabolic role of PanZ (although the activation is also clearly essential). To activate PanD, the PanZ.CoA complex needs to interact with PanD only once; the activation is irreversible and thus can still occur, even at low CoA concentrations. In contrast, inhibition of catalysis requires accumulation of PanZ.RCoA to form a substantial concentration of the inhibited ADC-PanZ.RCoA complex. Full inhibition of ADC will therefore only occur at sufficiently high CoA concentrations. Given that CoA concentrations in the cell can be as high as 4 mM (Bennett et al., 2009) compared with an affinity of 2 μM for the PanZ.CoA complex, we anticipate that activated ADC actually exists predominantly as the inhibited ADC-PanZ.RCoA complex in the cell. We suggest that this is the principal regulatory point for pantothenate biosynthesis and, by extension, de novo CoA

biosynthesis, and is sufficient to globally negatively regulate pantothenate biosynthesis (Figure 7D).

Regulation of biosynthesis by terminal metabolites via allosteric inhibition is well characterized; the involvement of a second protein is, however, unusual. PanZ was first identified as a putative *N*-acetyltransferase and, while there is no indication that it does not also carry out this enzymatic activity in vivo, ADC is not its substrate (Stuecker et al., 2012b). Acetylation is an ubiquitous post-translational modification in Gram-negative bacteria, and numerous such uncharacterized acetyltransferases exist (Jones and O'Connor, 2011). The limited phylogenetic distribution of PanZ (and the absolute requirement for it in only a small subset of bacteria) suggests to us that PanZ was first recruited to regulate catalysis by ADC in response to CoA levels, independent of its acetylation activity, before becoming essential for the activation of ADC.

The PanD-PanZ interaction provides the first regulatory mechanism for the pantothenate biosynthetic pathway in *E. coli*. The biosynthetic pathways for many of the other B vitamins are tightly regulated by mechanisms such as conserved riboswitches, despite the apparent lack of a significant fitness cost for vitamin overproduction. No equivalent regulatory mechanism for pantothenate has previously been identified. Here we have shown that in the case of pantothenate biosynthesis, this regulation is instead provided by this pantothenate metabolite-binding protein. We further suggest that regulation of catalysis by such metabolite-binding proteins could be a widespread phenomenon in other biosynthetic pathways and that many other such regulatory systems await discovery.

SIGNIFICANCE

Biosynthesis of many vitamins in bacteria is tightly regulated, yet no evidence for regulation of pantothenate biosynthesis has been reported. Here, we report the structure of the complex formed between the zymogen of aspartate decarboxylase, PanD, and its activating factor PanZ. Formation of this complex is dependent on the presence of CoA, the cofactor derived from pantothenate. The reported structure reveals the structural basis for the CoA dependence of interaction and provides a model for how formation of a PanD-PanZ.RCoA complex stimulates activation of the zymogen to form the activated enzyme. The involvement of CoA in this process suggested a paradoxical situation, in which an increase in concentration of a cofactor would stimulate its own biosynthesis. Further investigation, however, revealed that PanZ.RCoA can, in fact, act to inhibit catalysis by the activated enzyme. This suggests that the physiological role of PanZ is 2-fold: to catalyze the generation of active enzyme and to regulate subsequent activity by this enzyme. We suggest that this activity may have evolved by recruitment of an existing CoA-utilizing enzyme into a regulatory role, and that such regulatory protein-protein interactions may operate in other biosynthetic pathways.

EXPERIMENTAL PROCEDURES

Full details of protein expression, X-ray crystallography, NMR, SAXS analysis complementation assays, and ITC can be found in Supplemental Information.

Protein Crystallization and Structure Solution

Protein complexes were prepared with a 10:11 ratio of PanD to PanZ at a total protein concentration of 9–11 mg ml^{-1}, and a 2-fold molar excess (with respect to PanZ) of AcCoA added. Crystals were obtained in 20% (w/v) polyethylene glycol 3350, 0.1 M bis-Tris propane (pH 7.4), and 0.2 M potassium thiocyanate. Following data collection, X-ray data were indexed and integrated in space group *I4* using iMosflm (Leslie and Powell, 2007), and scaled and merged using Aimless (Evans and Murshudov, 2013). Phasing was carried out by molecular replacement using Molrep (Vagin and Teplyakov, 1997) and the coordinates from PDB 4AZD (ADC) and 2K5T (PanZ). The solutions were subjected to iterative rounds of manual rebuilding and refinement using Coot (Emsley et al., 2010) and Refmac5 (Murshudov et al., 2011).

SAXS

SAXS data were collected on beamline 4-2 of the Stanford Synchrotron Radiation Light Source (SSRL). PanD(T57V) and PanZ were mixed together with AcCoA in a 1:1:2 ratio, respectively. The data were integrated with SASTool and examined with PRIMUS (Konarev et al., 2003). Higher-order dimeric species were identified using OLIGOMER (Petoukhov et al., 2012), and a simulated scattering profile computed by FoXS (Schneidman-Duhovny et al., 2013) was subtracted from the SAXS data to yield the scattering for the isolated complex. Ab initio shape reconstructions were generated by DAMMIF (Franke and Svergun, 2009) using *P4* symmetry and averaged with DAMAVER (Volkov and Svergun, 2003). CORAL (Petoukhov et al., 2012) was used determine the positions of the crystallographically disordered residues. SUPCOMB (Kozin and Svergun, 2001) was used to align the high-resolution model with the envelope reconstruction.

ITC

ITC experiments were performed using a Microcal iTC200 (GE) or Microcal VP-ITC (GE) thermostated at 25 °C as described in the Supplemental Information. For global fitting, data were integrated using NITPIC (Keller et al., 2012) before global fitting to a one-site binding model in SEDPHAT (Houtman et al., 2007).

NMR

All protein NMR experiments were run at 25 °C in 50 mM Tris-HCl (pH 7.5), 0.1 M NaCl, and 0.1 mM DTT. ^{1}H-^{15}N HSQC spectra were obtained using a 500 MHz Varian Inova spectrometer. A spectral window of 8,000 Hz for ^{1}H (2,048 complex points) and 1,800 Hz for ^{15}N (92 increments) was used with 96 scans per increment. A protein concentration of 0.2 mM PanZ was used with a total acquisition time of 6 hr. All data were processed with NMRPipe (Delaglio et al., 1995) and analyzed using NMRView (Johnson and Blevins, 1994).

ACCESSION NUMBERS

The atomic coordinates and structure factors have been deposited in the Protein Data Bank, www.pdb.org under PDB ID 4CRZ (PanD(T57V)-PanZ.AcCoA) and 4CS0 (PanD(S25A)-PanZ.AcCoA).

SUPPLEMENTAL INFORMATION

Supplemental Information includes Supplemental Materials and Methods, seven figures, and one table and can be found with this article online at http://dx.doi.org/10.1016/j.chembiol.2015.03.017.

AUTHOR CONTRIBUTIONS

M.E.W, A.R.P., D.C.F.M., and H.N. conceived the project. D.C.F.M., J.A.G., C.B., S.N., and V.P. generated expression constructs, purified protein, and undertook crystallization experiments. D.C.F.M., V.P., and A.R.P. collected and refined crystallographic data. A.R.P., T.D.G., and E.H.S. conducted and analyzed SAXS experiments. S.N. undertook microbial growth experiments. D.C.F.M. and M.E.W. undertook enzyme kinetics assays. C.B., G.S.T., and A.P.K. performed and analyzed NMR experiments. M.E.W., J.A.G., and D.C.F.M. conducted and analyzed binding experiments. D.C.F.M., A.R.P., H.N., and M.E.W. wrote the paper.

ACKNOWLEDGMENTS

We would like to thank Chi Trinh and Stuart Warriner for scientific discussions and critical suggestions regarding this article. Personal funding to D.C.F.M., C.B., and J.A.G., and funding for the X-ray facility, NMR facility, and ITC was provided by the Wellcome Trust (096684/Z/11/Z, 099759/Z/12/Z, 093792/Z/10/Z, 094232/Z/10/Z, 062164). Collaborative funding to A.R.P., T.D.G., and E.H.S. was provided by the BBSRC (BB/I025247/1). SAXS data were collected at the SSRL beamline 4-2.

Received: February 18, 2015
Revised: March 24, 2015
Accepted: March 25, 2015
Published: April 23, 2015

REFERENCES

Albert, A., Dhanaraj, V., Genschel, U., Khan, G., Ramjee, M.K., Pulido, R., Sibanda, B.L., von Delft, F., Witty, M., Blundell, T.L., et al. (1998). Crystal structure of aspartate decarboxylase at 2.2 Å resolution provides evidence for an ester in protein self-processing. Nat. Struct. Mol. Biol. *5*, 289–293.

Bale, S., Lopez, M.M., Makhatadze, G.I., Fang, Q., Pegg, A.E., and Ealick, S.E. (2008). Structural basis for putrescine activation of human S-adenosylmethionine decarboxylase. Biochemistry *47*, 13404–13417.

Bennett, B.D., Kimball, E.H., Gao, M., Osterhout, R., Van Dien, S.J., and Rabinowitz, J.D. (2009). Absolute metabolite concentrations and implied enzyme active site occupancy in *Escherichia coli*. Nat. Chem. Biol. *5*, 593–599.

Buller, A.R., Freeman, M.F., Wright, N.T., Schildback, J.F., and Townsend, C.A. (2012). Insights into cis-autoproteolysis reveal a reactive state formed through conformational rearrangement. Proc. Natl. Acad. Sci. USA *109*, 2308–2313.

Cort, J.R., Yee, A., Arrowsmith, C.H., and Kennedy, M.A. (2009). Cofactor Optimization. http://www.nmr2.buffalo.edu/nesg.wiki/Cofactor_optimization.

Delaglio, F., Grzesiek, S., Vuister, G.W., Zhu, G., Pfeifer, J., and Bax, A. (1995). NMRPipe: a multidimensional spectral processing system based on UNIX pipes. J. Biomol. NMR *6*, 277–293.

Emsley, P., Lohkamp, B., Scott, W.G., and Cowtan, K. (2010). Features and development of Coot. Acta Crystallogr. D Biol. Crystallogr. *66*, 486–501.

Evans, P.R., and Murshudov, G.N. (2013). How good are my data and what is the resolution? Acta Crystallogr. D Biol. Crystallogr. *69*, 1204–1214.

Farrar, C.E., Siu, K.K., Howell, P.L., and Jarrett, J.T. (2010). Biotin synthase exhibits burst kinetics and multiple turnovers in the absence of inhibition by products and product-related biomolecules. Biochemistry *49*, 9985–9996.

Franke, D., and Svergun, D.I. (2009). DAMMIF, a program for rapid ab-initio shape determination in small-angle scattering. J. Appl. Cryst. *42*, 342–346.

Gelfman, C.M., Copeland, W.C., and Robertus, J.D. (1991). Site-directed alteration of four active-site residues of a pyruvoyl-dependent histidine decarboxylase. Biochemistry *30*, 1057–1062.

Grose, J.H., Bergthorsson, U., and Roth, J.R. (2005). Regulation of NAD synthesis by the trifunctional NadR protein of *Salmonella enterica*. J. Bacteriol. *187*, 2774–2782.

Houtman, J.C., Brown, P.H., Bowden, B., Yamaguchi, H., Appella, E., Samelson, L.E., and Schuck, P. (2007). Studying multisite binary and ternary protein interactions by global analysis of isothermal titration calorimetry data in SEDPHAT: application to adaptor protein complexes in cell signaling. Protein Sci. *16*, 30–42.

Johannson, D.G., Wallin, G., Sandberg, A., Macao, B., Åqvist, J., and Härd, T. (2009). Protein autoproteolysis: conformation strain linked to the rate of peptide cleavage by the pH dependence of the N→O acyl shift. J. Amer. Chem. Soc. *131*, 9475–9477.

Johnson, B., and Blevins, R. (1994). NMR view: A computer program for the visualization and analysis of NMR data. J. Biomol. NMR *4*, 603–614.

Jones, J.D., and O'Connor, C.D. (2011). Protein acetylation in prokaryotes. Proteomics *11*, 3012–3022.

Keller, S., Vargas, C., Zhao, H., Piszczek, G., Brautigam, C.A., and Schuck, P. (2012). High-precision isothermal titration calorimetry with automated peak-shape analysis. Anal. Chem. *84*, 5066–5073.

Kim, J.K., Yang, I.S., Shin, H.Y., Cho, K.J., Ryu, E.K., Kim, S.H., Park, S.S., and Kim, K.H. (2006). Insight into autoproteolytic activation from the structure of cephalosporin acylase: A protein with two proteolytic chemistries. Proc. Natl. Acad. Sci. USA *103*, 1732–1737.

Konarev, P.V., Volkov, V.V., Sokolova, A.V., Koch, M.H.J., and Svergun, D.I. (2003). PRIMUS: a Windows PC-based system for small-angle scattering data analysis. J. Appl. Cryst. *36*, 1277–1282.

Konst, P.M., Franssen, M.C.R., Scott, E.L., and Sanders, J.P.M. (2009). A study on the applicability of L-aspartate α-decarboxylase in the biobased production of nitrogen containing chemicals. Green. Chem. *11*, 1646–1652.

Kozin, M.B., and Svergun, D.I. (2001). Automated matching of high- and low-resolution structural models. J. Appl. Cryst. *34*, 33–41.

Leslie, A.W., and Powell, H. (2007). Processing diffraction data with mosflm. In Evolving Methods for Macromolecular Crystallography, R. Read and J. Sussman, eds. (Springer), pp. 41–51.

Levitin, F., Stern, O., Weiss, M., Gil-Henn, C., Ziv, R., Prolocimer, Z., Smorodinsky, N.I., Rubinstein, D.B., and Wreschner, D.H. (2005). The MUC1 SEA module is a self-cleaving domain. J. Biol. Chem. *280*, 33374–33386.

McDonald, J.P., Frank, E.G., Levine, A.S., and Woodgate, R. (1998). Intermolecular cleavage by UmuD-like mutagenesis proteins. Proc. Natl. Acad. Sci. USA *95*, 1478–1483.

Monteiro, D.C., Rugen, M.D., Shepherd, D., Nozaki, S., Niki, H., and Webb, M.E. (2012). Formation of a heterooctameric complex between aspartate alpha-decarboxylase and its cognate activating factor, PanZ, is CoA-dependent. Biochem. Biophys. Res. Commun. *426*, 350–355.

Murshudov, G.N., Skubak, P., Lebedev, A.A., Pannu, N.S., Steiner, R.A., Nicholls, R.A., Winn, M.D., Long, F., and Vagin, A.A. (2011). REFMAC5 for the refinement of macromolecular crystal structures. Acta Crystallogr. D Biol. Crystallogr. *67*, 355–367.

Nozaki, S., Webb, M.E., and Niki, H. (2012). An activator for pyruvoyl-dependent l-aspartate α-decarboxylase is conserved in a small group of the γ-proteobacteria including Escherichia coli. Microbiologyopen *1*, 298–310.

Paulus, H. (2000). Protein splicing and related forms of protein autoprocessing. Annu. Rev. Biochem. *69*, 447–496.

Pegg, A.E. (2009). S-Adenosylmethionine decarboxylase. Essays Biochem. *46*, 25–46.

Petoukhov, M.V., Franke, D., Shkumatov, A.V., Tria, G., Kikhney, A.G., Gajda, M., Gorba, C., Mertens, H.D.T., Konarev, P.V., and Svergun, D.I. (2012). New developments in the ATSAS program package for small-angle scattering data analysis. J. Appl. Cryst. *45*, 342–350.

Ramachandran, G.N., Ramakrishnan, C., and Sasisekharan, V. (1963). Stereochemistry of polypeptide chain configurations. J. Mol. Biol. *7*, 95–99.

Ramjee, M.K., Genschel, U., Abell, C., and Smith, A.G. (1997). *Escherichia coli* L-aspartate alpha-decarboxylase: preprotein processing and observation of reaction intermediates by electrospray mass spectrometry. Biochem. J. *323*, 661–669.

Rock, C.O., Park, H.-W., and Jackowski, S. (2003). Role of feedback regulation of pantothenate kinase (CoaA) in control of coenzyme A levels in Escherichia coli. J. Bacteriol. *185*, 3410–3415.

Schelp, E., Worley, S., Monzingo, A.F., Ernst, S., and Robertus, J.D. (2001). pH-induced structural changes regulate histidine decarboxylase activity in Lactobacillus 30a. J. Mol. Biol. *306*, 727–732.

Schmitzberger, F., Kilkenny, M.L., Lobley, C.M., Webb, M.E., Matak-Vinkovic, D., Witty, M., Chirgadze, D.Y., Smith, A.G., Abell, C., and Blundell, T. (2003). Structural constraints on protein self-processing in L-aspartate-alpha-decarboxylase. EMBO J. *22*, 6193–6204.

Schneidman-Duhovny, D., Hammel, M., Tainer, J.A., and Sali, A. (2013). Accurate SAXS profile computation and its assessment by contrast variation experiments. Biophys. J. *105*, 962–974.

Schuiki, I., and Daum, G. (2009). Phosphatidylserine decarboxylases, key enzymes of lipid metabolism. IUBMB Life *61*, 151–162.

Snell, E.E. (1986). Pyruvoyl-dependent histidine decarboxylase from lactobacillus 30a: purification and properties. Methods Enzymol. *122*, 128–135.

Stanley, B.A., Shantz, L.M., and Pegg, A.E. (1994). Expression of mammalian S-adenosylmethionine decarboxylase in *Escherichia coli*. Determination of sites for putrescine activation of activity and processing. J. Biol. Chem. *269*, 7901–7907.

Stuecker, T.N., Hodge, K.M., and Escalante-Semerena, J.C. (2012a). The missing link in coenzyme A biosynthesis: PanM (formerly YhhK), a yeast GCN5 acetyltransferase homologue triggers aspartate decarboxylase (PanD) maturation in *Salmonella enterica*. Mol. Microbiol. *84*, 608–619.

Stuecker, T.N., Tucker, A.C., and Escalante-Semerena, J.C. (2012b). PanM, an acetyl-coenzyme A sensor required for maturation of L-aspartate decarboxylase (PanD). MBio *3*, e00158–12.

Todd, M.J., and Gomez, J. (2001). Enzyme kinetics determined using calorimetry: a general assay for enzyme activity? Anal. Biochem. *296*, 179–187.

Tolbert, W.D., Graham, D.E., White, R.H., and Ealick, S.E. (2003a). Pyruvoyl-dependent arginine decarboxylase from *Methanococcus jannaschii*: crystal structures of the self-cleaved and S53A proenzyme forms. Structure *11*, 285–294.

Tolbert, W.D., Zhang, Y., Cottet, S.E., Bennett, E.M., Ekstrom, J.L., Pegg, A.E., and Ealick, S.E. (2003b). Mechanism of human S-adenosylmethionine decarboxylase proenzyme processing as revealed by the structure of the S68A mutant. Biochemistry *42*, 2386–2395.

Trip, H., Mulder, N.L., Rattray, F.P., and Lolkema, J.S. (2011). HdcB, a novel enzyme catalysing maturation of pyruvoyl-dependent histidine decarboxylase. Mol. Microbiol. *79*, 861–871.

Vagin, A., and Teplyakov, A. (1997). MOLREP: an automated program for molecular replacement. J. Appl. Cryst. *30*, 1022–1025.

van Poelje, P.D., and Snell, E.E. (1990). Pyruvoyl-dependent enzymes. Annu. Rev. Biochem. *59*, 29–59.

Vetting, M.W., de Carvalho, L.P.S., Yu, M., Hege, S.S., Magnet, S., Roderick, S.L., and Blanchard, J.S. (2005). Structure and functions of the GNAT superfamily of acetyltransferases. Arch. Biochem. Biophys. *433*, 212–226.

Volkov, V.V., and Svergun, D.I. (2003). Uniqueness of ab initio shape determination in small-angle scattering. J. Appl. Cryst. *36*, 860–864.

Wang, M., Weiss, M., Simonovic, M., Haertinger, G., Schrimpf, S.P., Hengartner, M.O., and von Mering, C. (2012). PaxDb, a database of protein abundance averages across all three domains of life. Mol. Cell Proteomics *11*, 492–500.

Webb, M.E., Smith, A.G., and Abell, C. (2004). Biosynthesis of pantothenate. Nat. Prod. Rep. *21*, 695–721.

Webb, M.E., Lobley, C.M.C., Soliman, F., Kilkenny, M.L., Smith, A.G., Blundell, T.L., and Abell, C. (2012). Structure of Escherichia coli aspartate [alpha]-decarboxylase Asn72Ala: probing the role of Asn72 in pyruvoyl cofactor formation. Acta Crystallogr. Sect F Struct. Biol. Cryst. Commun. *68*, 414–417.

Webb, M.E., Yorke, B.A., Kershaw, T., Lovelock, S., Lobley, C.M.C., Kilkenny, M.L., Smith, A.G., Blundell, T.L., Pearson, A.R., and Abell, C. (2014). Threonine 57 is required for the post-translational activation of *E. coli* aspartate a-decarboxylase. Acta Crystallogr. D Biol. Crystallogr. *70*, 1166–1172.

Williamson, J.M., and Brown, G.M. (1979). Purification and properties of L-aspartate alpha-decaboxylase, an enzyme that catalyses the formation of beta-alanine in *Escherichia coli*. J. Biol. Chem. *254*, 8074–8082.

Winkler, W.C., and Breaker, R.R. (2005). Regulation of bacterial gene expression by riboswitches. Annu. Rev. Microbiol. *59*, 487–517.

Yun, M., Park, C.-G., Kim, J.-Y., Rock, C.O., Jackowski, S., and Park, H.-W. (2000). Structural basis for the feedback regulation of *Escherichia coli* pantothenate kinase by coenzyme A. J. Biol. Chem. *275*, 28093–28099.

Chemistry & Biology, Volume *22*

Supplemental Information

The Structure of the PanD/PanZ Protein Complex Reveals Negative Feedback Regulation of Pantothenate Biosynthesis by Coenzyme A

Diana C.F. Monteiro, Vijay Patel, Christopher P. Bartlett, Shingo Nozaki, Thomas D. Grant, James A. Gowdy, Gary S. Thompson, Arnout P. Kalverda, Edward H. Snell, Hironori Niki, Arwen R. Pearson, and Michael E. Webb

Supplemental figures

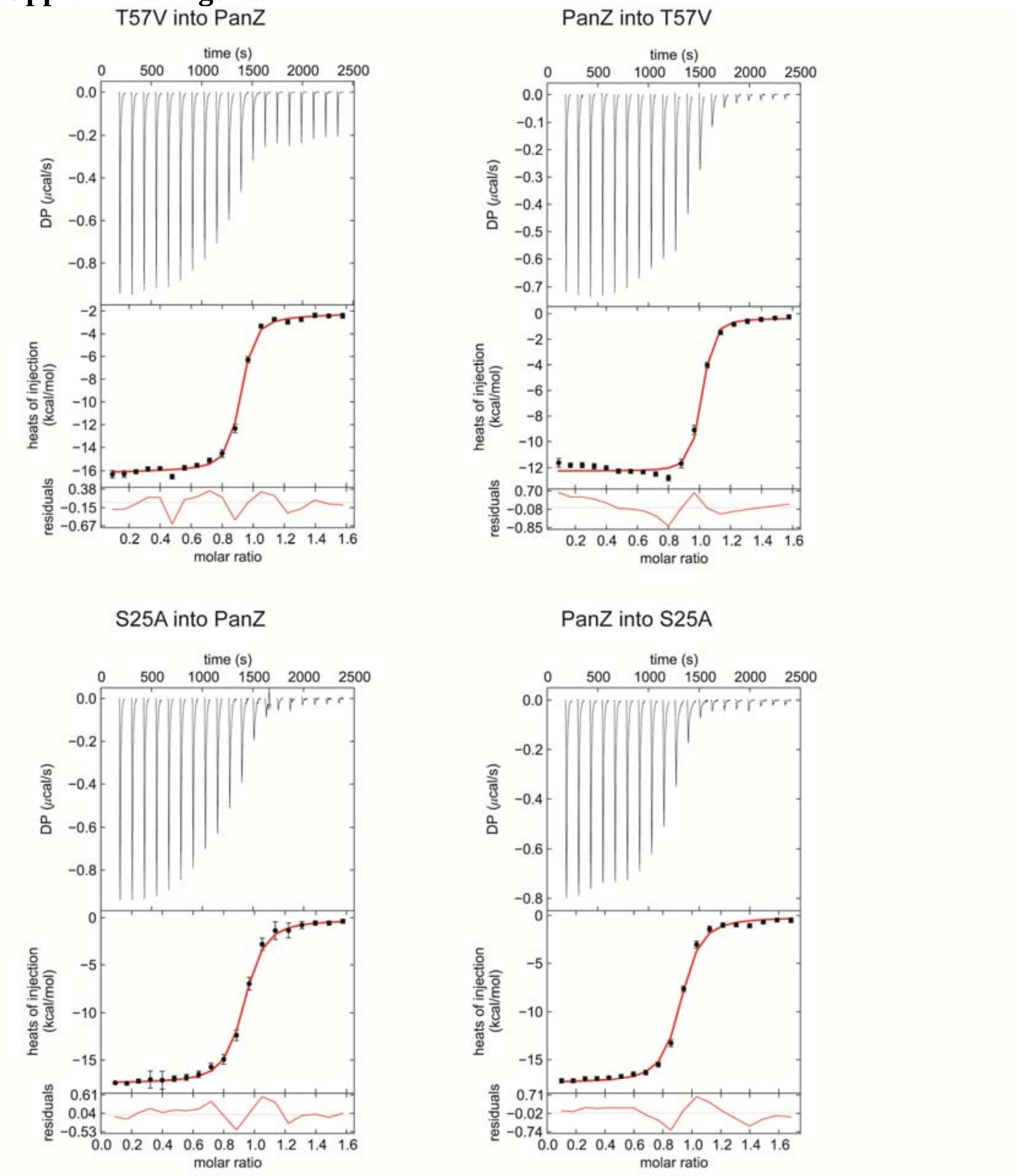

Mutant	Log K_a	K_d /nM	ΔH /kcal mol^{-1}	ΔG /kcal mol^{-1}	ΔS /cal mol^{-1} K^{-1}
T57V	7.457 ± 0.045	34.9 ± 3.6	-11.89 ± 0.07	-10.16	-5.8
S25A	6.803 ± 0.015	157.2 ± 5.5	-17.20 ± 0.03	-9.27	-26.6

Figure S1, related to figures 2 and 4 Global analysis of interaction between PanZ and PanD by ITC. **A** Pairwise titration of 263 μM PanD(T57V) into 35 μM PanZ in the presence of 400 μM AcCoA and 263 μM PanZ into 35 μM PanD(T57V) in the presence of 400 μM AcCoA. **B** Pairwise titration of 257 μM PanD(S25A) into 32 μM PanZ in the presence of 394 μM AcCoA and 263 μM PanZ into 35 μM PanD(S25A) in the presence of 400 μM AcCoA. **C** Thermodynamic parameters for interaction obtained by global fitting of both sets of pairwise titrations. Quoted errors are standard errors (n=38) based on direct determination of the parameter values corresponding to critical changes in χ^2 corresponding to a 66% confidence interval.

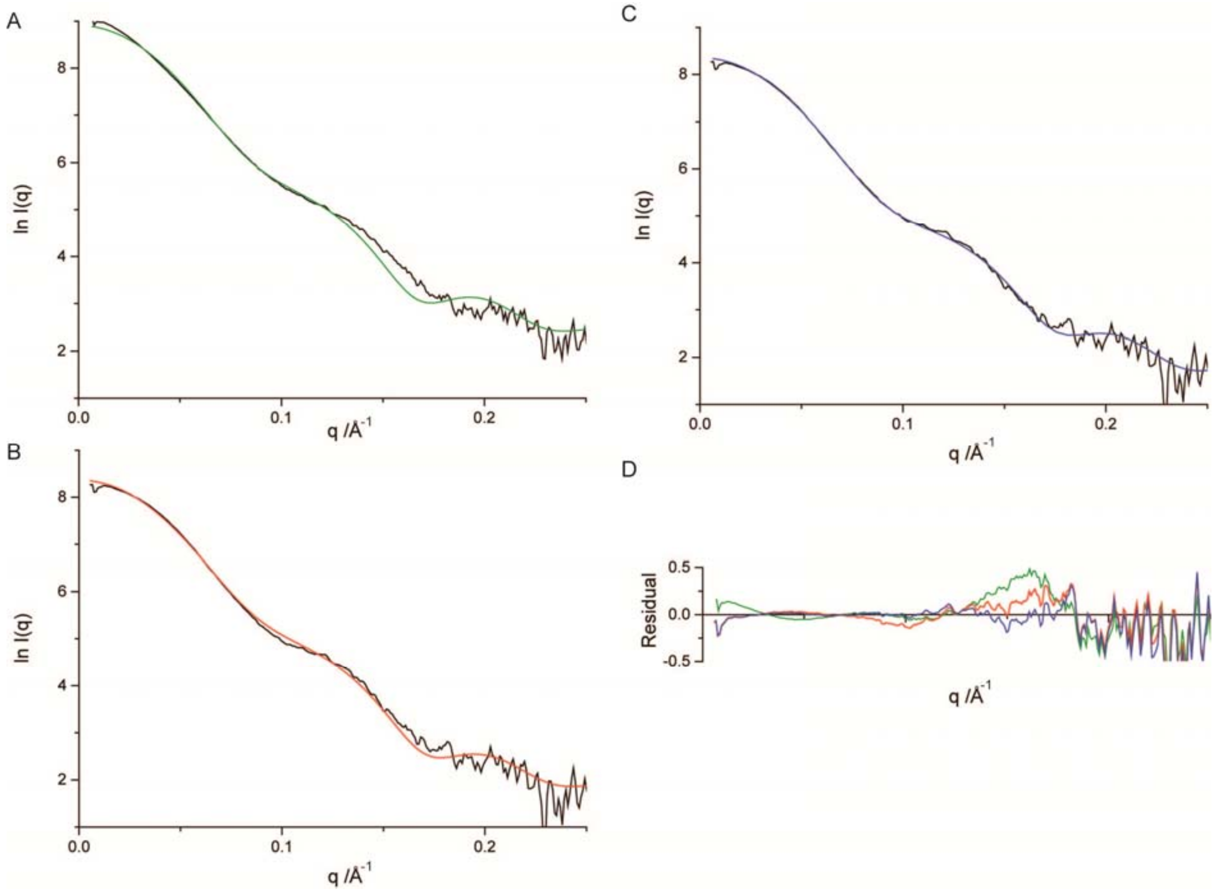

Figure S2, related to figure 2 SAXS data and fitting. **A** Raw SAXS data for the PanD(T57V)-PanZ.AcCoA complex (black) compared with predicted data for the crystallographically resolved heterooctameric complex (green). **B** Inclusion of a population of dimers of heterooctamers leads to an improved fit (red) compared to the monomer. **C** Subsequent inclusion of the eight *C*- and *N*-terminal affinity purification tags using a coarse-grained model leads to a further improved fit (blue). **D** Residuals from three sequential rounds of data fitting: heterooctamer (green), inclusion of dimer of heterooctamers (red), inclusion of affinity tags (blue).

A

B

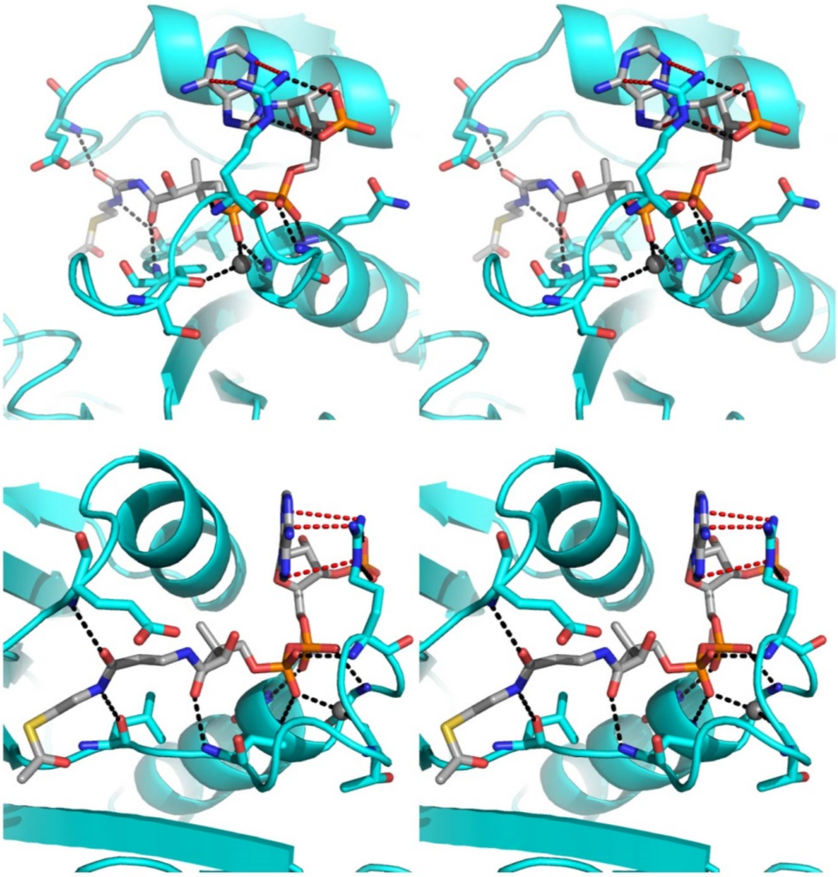

Figure S3, related to figure 3 Hydrogen-bonding interactions in the PanZ AcCoA binding pocket. **A** Front view of the AcCoA binding pocket (in stereo), showing hydrogen-bonds (black dashed lines) and cation-π interactions (red dashed lines) between AcCoA, Mg^{2+} and PanZ. All interacting residues, with the exception of Glu23, Gly78 and Gln79 are located in the PanZ P-loop. **B** Side view of the same binding pocket and interactions (also in stereo).

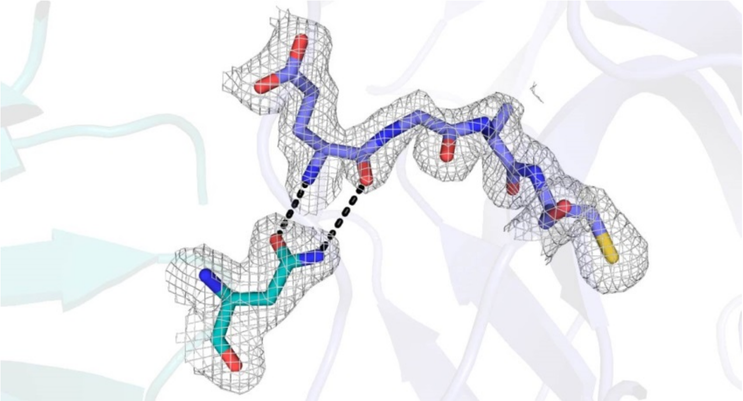

Figure S4, related to figure 5

Sample electron density for the PanD(S25A).PanZ.AcCoA complex showing conformation of the activatable loop and bidentate hydrogen bonding interactions between PanZ-Asn45 and the backbone amide of PanD-Glu23. The $2F_0$-F_c electron density map is contoured at 1 r.m.s.d.

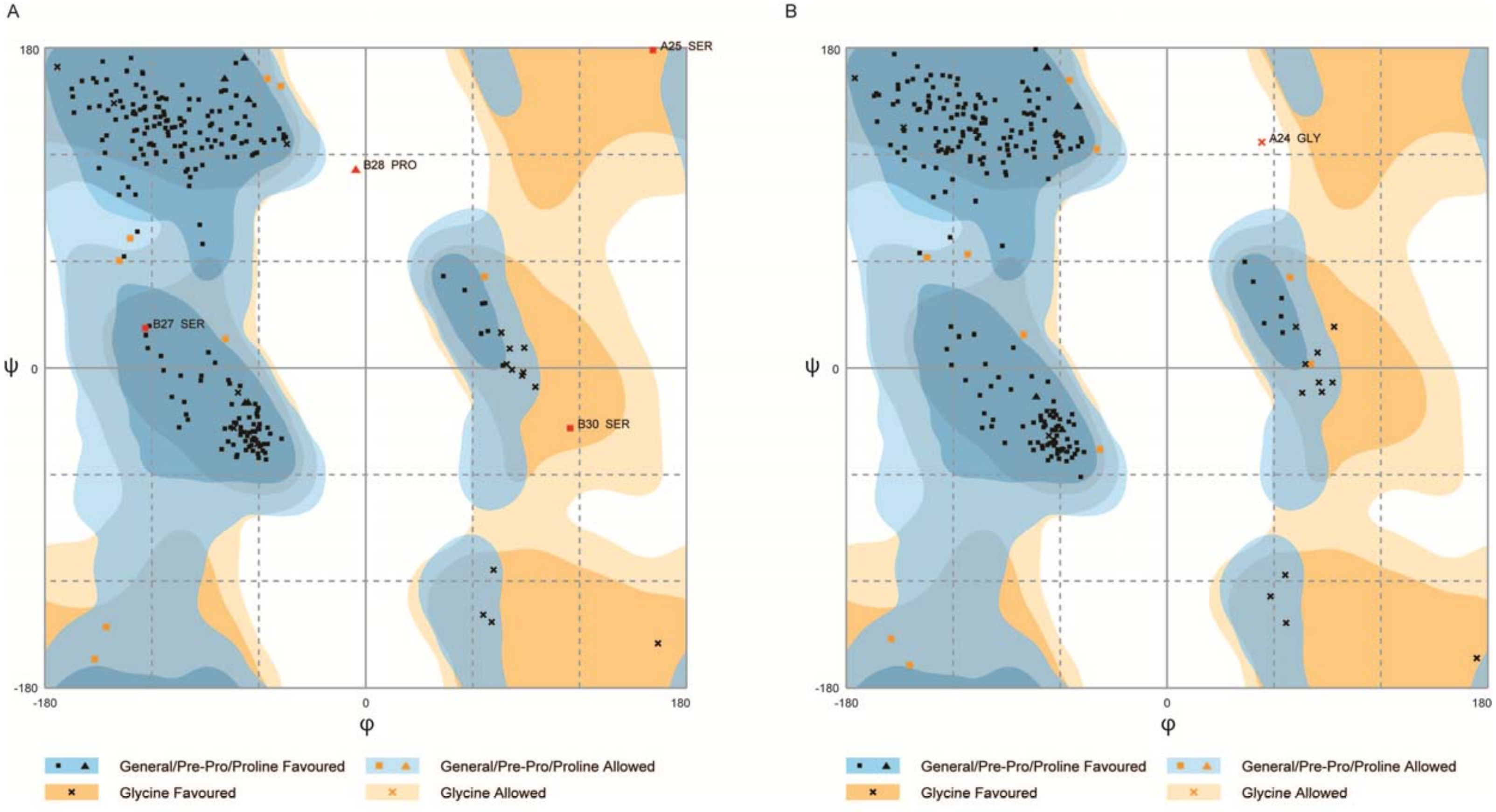

A

Number of residues in favoured region (~98.0% expected) : 236 (95.2%)
Number of residues in allowed region (~2.0% expected) : 8 (3.2%)
Number of residues in outlier region : 4 (1.6%)

B

Number of residues in favoured region (~98.0% expected) : 236 (95.5%)
Number of residues in allowed region (~2.0% expected) : 10 (4.0%)
Number of residues in outlier region : 1 (0.4%)

Figure S5, related to figure 5 Ramachandran plots for the PanD-PanZ complex. **A** Ramachandran plot for the PanD(T57V)-PanZ.AcCoA complex. Residue PanD-Ser25 (A25 SER) lies in a outlier region. (Residues B27-B30 correspond to a poorly defined region of PanZ) **B** Ramachandran plot for the PanD(S25A)-PanZ.AcCoA complex. Residue PanD-Gly24 lies in an outlier region (Lovell et al., 2003). Ramachandran plots were generated using RAMPAGE.

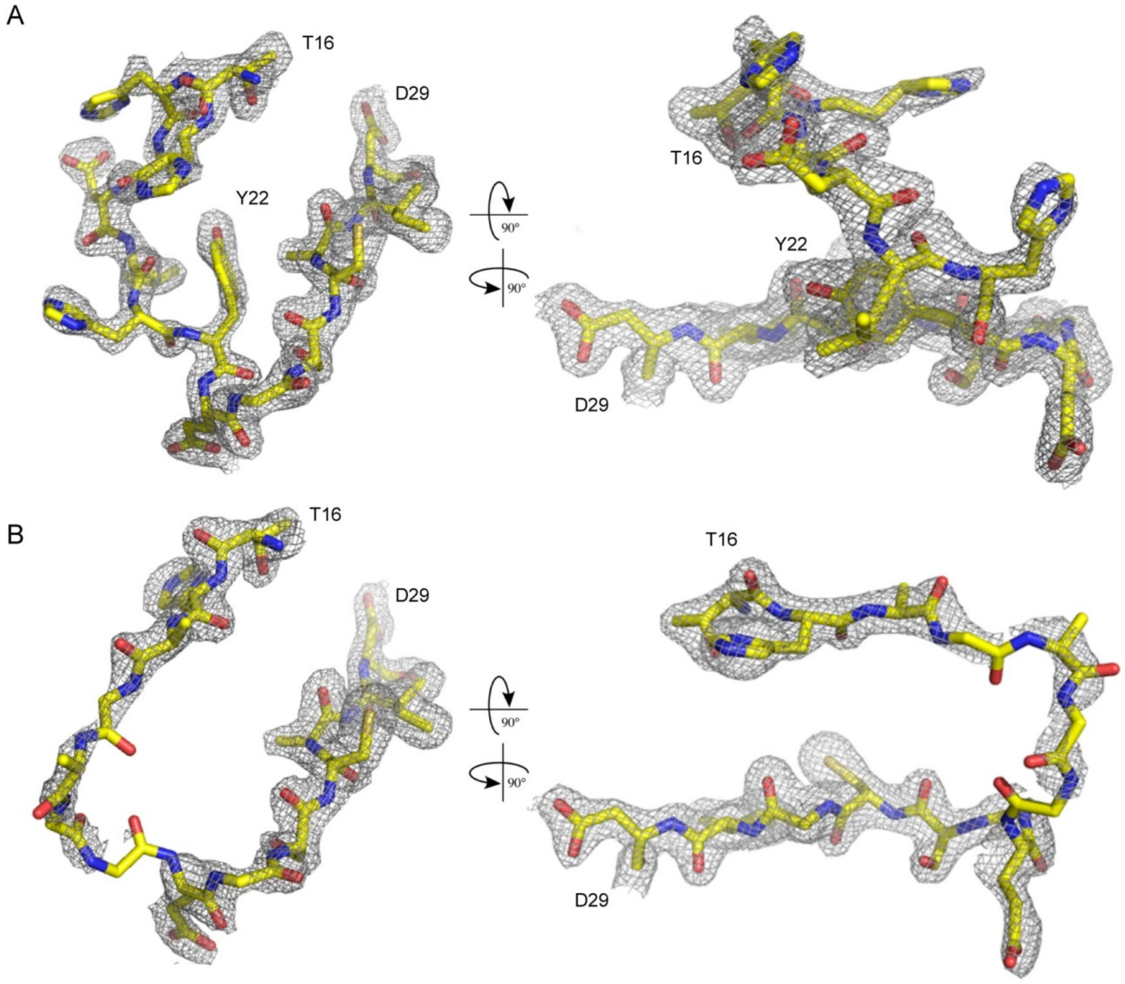

Figure S6, related to figure 5 and 7 The different processing loop conformations adopted by the two pADC zymogen protomers in the asymmetric unit of the structure solved previously by Schmitzberger *et al.* (PDB 1PPY (Schmitzberger et al., 2003)) Residues Thr16-Asp29 are represented as sticks and the accompanying $2F_0$-F_c electron density map is contoured at 1 r.m.s.d. **A** The non-activatable conformation observed in conformer A, showing a well defined electron density map throughout the entire loop. **B** The activatable conformation observed in protomer B, showing a much less well defined electron density map in the processing region, with the sidechain of Tyr22 unresolved. In the absence of PanZ, this conformation is disfavoured and only the electron-density for residues Thr16-Ala18 (which form a new β-sheet) is well defined. Consequently once the β-sheet has formed the remainder of the loop does not adopt one single conformation, explaining the poorly defined electron density.

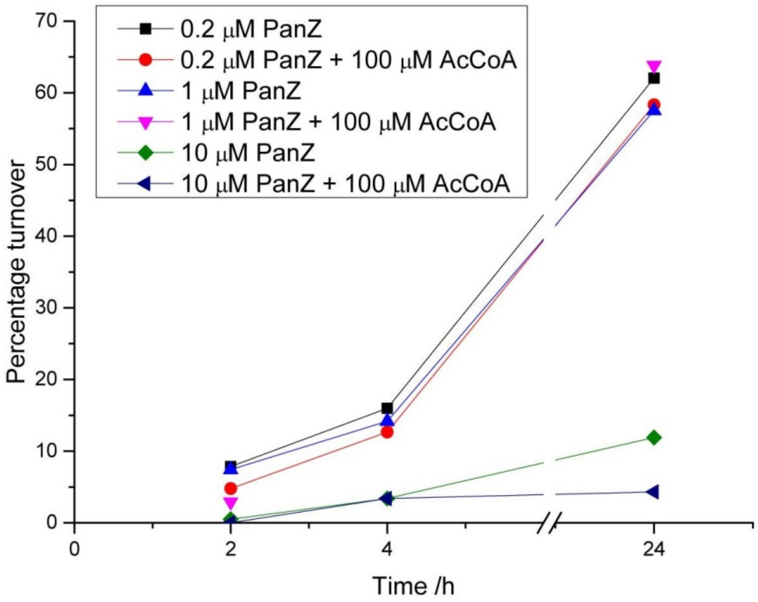

Figure S7, related to figure 6 ADC inhibition by PanZ. The percentage turnover of L-aspartate by ADC over time was monitored using 500 MHz ^{1}H NMR. Increase in the concentration of PanZ in solution leads to decreased turnover.

Table S1, related to figure 2 Data collection and refinement statistics (molecular replacement)

	pADC(T57V)-PanZ	pADC(S25A)-PanZ
**Data collection**		
Space group	I4	I4
Cell dimensions		
a, *b*, *c* (Å)	86.3, 86.3, 80.9	86.3, 86.3, 80.8
α, β, γ (°)	90.0, 90.0, 90.0	90.0, 90.0, 90.0
Resolution (Å)	33.7-1.70 (1.74-1.70)*	33.7-2.10 (2.16-2.10)*
R_{sym} or R_{merge}	3.7 (39.8)	18.1 (62.1)
R_{pim}	3.6 (36.6)	15.5 (51.7)
$I / \sigma I$	13.3 (2.2)	5.7 (1.8)
Completeness (%)	99.7 (99.7)	99.5 (99.8)
Redundancy	2.4 (2.3)	3.2 (3.1)
**Refinement**		
PDB ID	4CRZ	4CS0
Resolution (Å)	29.52 (1.70)	33.7 (2.10)
No. reflections	30609	16351
R_{work} / R_{free}	14.46 / 17.52	17.21 / 23.69
No. atoms		
Protein	2064	2021
Ligand/ion	65	62
Water	145	62
B-factors		
Protein (main chain)	20.35	24.29
Protein (side chain)	25.99	28.57
Ligand/ion	20.35	28.47
Water	32.45	26.86
R.m.s. deviations		
Bond lengths (Å)	0.023	0.019
Bond angles (°)	2.33	2.09

*All datasets were collected from a single crystal, values in parentheses are for highest-resolution shell.

Supplemental Materials and Methods:

Protein expression and purification

The coding sequence for C-terminally hexahis-tagged PanZ was subcloned into the pET28a backbone from pBAD24 by PCR to allow protein overexpression by auto-induction of T7 RNA polymerase. The coding sequence for PanZ, including the ribosome-binding site, was amplified by PCR using the primers PanZ-F (5′-GTGATGCTAGCAGGAGGAATTCC-3′) and PanZ-R (5′-GAATCTCGAGACCGCTACACTTCTCC-3′) from the pBAD24-PanZ construct described previously (Nozaki et al., 2012). The amplified product was digested (NheI and XhoI) before ligation into pET28a previously linearised with XbaI and XhoI. The sequence of the construct was confirmed by DNA sequencing (GATC).

His-tagged WT-ADC, PanD(T57V), PanD(S25A), PanZ and PanZ(N45A) were overexpressed using an *E. coli ΔpanD ΔpanZ* (DE3) cell strain as described previously (Monteiro et al., 2012; Nozaki et al., 2012). For ITC activity assays, His-tagged WT-ADC was overexpressed using *E. coli* C41 (DE3). His-tagged WT-ADC, PanD(T57V) and PanD(S25A) were overexpressed from the vectors pRSETA-ADC-WT (Saldanha et al., 2001), pRSETA-ADC(T57V) (Webb et al., 2014), pRSETA-ADC(S25A) (Schmitzberger et al., 2003) and pET28a-PanZ using an autoinduction protocol (Studier, 2005). For ITC kinetic analysis, ADC was overexpressed from pRSETA-ADC-WT in *E. coli* C41 (DE3). PanZ(N45A) was overexpressed from the vector pBAD24-PanZ(N45A) using arabinose induction as described previously (Nozaki et al., 2012). Cells were isolated by centrifugation (10,000*g*, 15 min), resuspended in buffer A (50 mM potassium phosphate, 300 mM NaCl, pH 7.4) containing 10 mM imidazole, mechanically lysed using a Constant Systems cell disrupter (20 kpsi) and the lysate cleared by centrifugation (30,000*g*, 45 min). DNase I (Roche, ~0.5 mgL^{-1} culture) was added to the cleared lysate before application to a Ni-NTA agarose (Qiagen) column under gravity flow. The column was washed with 10 column volumes of wash buffer (Buffer A + 50 mM imidazole) before elution of protein fractions using elution buffer (Buffer A + 250 mM imidazole). Protein-containing fractions, which were identified by SDS–PAGE, were combined and concentrated using centrifugal concentration (Amicon, 10 kDa MWCO). Protein was purified to apparent homogeneity by isocratic elution from either a Hi-Load 26/60 or 16/60 Superdex 75 column (GE) mounted on an Akta Purifier FPLC system in 50 mM Tris–HCl, 100 mM NaCl, 0.1 mM DTT, pH 7.4 at 4 ºC. Protein identity was confirmed by electrospray mass spectrometry using a Bruker HCT-Ultra LC–MS system. Protein concentrations were determined by UV absorption at 280 nm using theoretical estimates for the protein concentration based on the primary sequence of the protein (ProtParam (Gasteiger et al., 2005): PanD ε_{280} 15470 M^{-1} cm^{-1}, PanZ ε_{280} 26470 M^{-1} cm^{-1}).

Protein crystallization and structure solution

Solutions of the protein complexes were prepared using a 10:11 ratio of PanD(T57V) to PanZ. The proteins were then concentrated to a total protein concentration of 9-11 mg mL^{-1} using centrifugal concentration (Amicon, 10 kDa MWCO) and a two-fold molar excess of AcCoA added. Sparse matrix screens using Crystal Screen, Crystal Screen 2, Index, and Salt RX (Hampton Research), and Wizard I and II (Emerald Bioscience) were set up using an Oryx 6 Douglas crystallization robot (Douglas Instruments) in MRC 96-well plates

(Molecular Dimensions) with 1.0 μL protein:1.0 μL mother liquor drops at 18 ºC. Crystals were obtained in 20% (*w*/*v*) polyethylene glycol (PEG) 3350, 0.1 M bis-tris propane pH 7.4, 0.2 M potassium thiocyanate. Optimisation was carried out by pH and salt concentration variation and crystals grown using the hanging drop vapour diffusion method. Crystals were mounted on MicroMeshes™ (Mitegen) and data collected at RT (298 K).

The PanD(T57V)-PanZ complex and PanD(S25A)-PanZ complex were collected at room temperature using the in-house source (MicroMax-007 HF microfocus rotating anode generator, 30 mA), λ = 1.5418 Å. 60 frames of 1° oscillation, 30 s exposure and full transmission were collected with to a maximum resolution of 1.64 Å and 2.1 Å respectively. Data were integrated in spacegroup *I4* using iMosflm (Leslie and Powell, 2007), and scaled and merged using Aimless (Evans and Murshudov, 2013). Phasing was carried out by molecular replacement using Molrep (Vagin and Teplyakov, 1997). PDB 4AZD was placed first, followed by 2K5T. One protomer of each (PanD and PanZ) are found in the asymmetric unit and these form the heterooctameric complex by symmetry. The solution was checked by removing the CoA ligand and searching for the corresponding positive signal on the F_o-F_c difference electron density map after 20 cycles of restrained refinement. The solutions were subjected to iterative rounds of manual rebuilding and refinement using Coot (Emsley et al., 2010) and refinement with Refmac5 (Murshudov et al., 2011).

Isothermal titration calorimetry

Isothermal titration calorimetry experiments were performed using a Microcal iTC200 (GE) or Microcal VP-ITC (GE) thermostatted at 25 °C. For the microcal iTC200, the ligand sample was loaded into the sample cell (200 μL) and the titrant into the sample syringe (40 μL). Each titration series consisted of a 0.4 μL injection followed by 19 injections of 2 μL. For the VP-ITC, the ligand sample was loaded into the sample cell (1.5 mL) and the titrant into the sample syringe (300 μL). Each titration series consisted of a 2 μL injection followed by 29 injections of 10 μL each. Samples were prepared in 50 mM Tris–HCl, 100 mM NaCl, pH 7.4, 0.1 mM DTT. For titrations in the presence of excess AcCoA, this was added to both the ligand and titrant sample to the same final concentration without any attempt to account for copurified CoA in the PanZ sample. Data were analysed in Origin 6.5. After baseline substraction data were fitted to a single site-binding model. For global fitting, data were integrated using NITPIC (Keller et al., 2012) before global fitting to a one-site binding model in SEDPHAT (Houtman et al., 2007). Errors in parameters obtained through global fitting were estimated using parameter values corresponding to critical χ^2 values on computed error surface projections using SEDPHAT.

For kinetic experiments, isothermal titration calorimetry experiments were performed using a Microcal VP-ITC (GE) thermostatted at 25 °C. Samples were prepared in 50 mM potassium phosphate pH 7.0. The enzyme sample was loaded into the sample cell (2 mL) and the substrate (monosodium L-aspartate, 25 mM) into the sample syringe (200 μL). Each titration series consisted of a 25 injections of 2 μL. The reaction rate was estimated based on the baseline change after each injection (using the measured enthalpy of reaction of 3.8 kcal mol^{-1} as a conversion factor.). All measurements were taken relative to a linear extrapolation of the pre-titration baseline.

Complementation assays

Wild-type cells (MG1655) harboring vector plasmid (pBAD24), Δ*panZ* cells (MG1655 ΔpanZ) harboring vector plasmid and Δ*panZ* cells harboring pBAD24-*panZ-his* were incubated overnight in 3 mL of L broth containing 50 μg/mL of ampicillin at 37 ºC. Cells were washed with M9 salt solution (6 g/L Na_2HPO_4, 3 g/L KH_2PO_4, 0.5 g/L NaCl, 1 g/L NH_4Cl) and resuspended in M9 salt solution to an adjusted OD_{600} of each cell suspension of 1.0. Cell suspensions were serially diluted in M9 salt solution at 10^{-2}, 10^{-3}, 10^{-4} 10^{-5} and 10^{-6} and 3 μL of each dilution was spotted on M9 agar plates containing 0.2% glucose and 50 μg/mL of ampicillin, M9 agar plates containing 0.2% of glucose, 0.5 mM of β-alanine, and 50 μg/mL of ampicillin, M9 agar plates containing 0.2% of L-(+)-arabinose and 50 μg/mL of ampicillin, and M9 agar plates containing 0.2% of L-(+)-arabinose, 0.5 mM of β-alanine, and 50 μg/mL of ampicillin. The M9 glucose plates and M9 arabinose plates were incubated at 37 ºC for 24 hours and for 48 hours, respectively and observed for formation of colonies.

SAXS

Small angle X-ray scattering data were collected on beamline 4-2 of the Stanford Synchrotron Radiation Lightsource (SSRL). Data were collected at a wavelength of λ = 1.3Å for eight consecutive two second exposures from three concentrations of protein ranging from 0.20 to 2.9 mg/mL. PanD(T57V) and PanZ were mixed together with AcCoA in a 1:1:2 ratio, respectively. The mixture was concentrated to c. 10 mg/mL using centrifugal concentration (Amicon, 10 kDa MWCO). The flowthrough was used as the buffer blank (50 mM Tris–HCl, 100 mM NaCl, 0.1 mM DTT, pH 7.4) and subtracted from the total protein solution scattering. The data were integrated with SASTool and examined with PRIMUS (Konarev et al., 2003). Analysis of eight consecutive time frames showed that no radiation damage took place over the course of the experiment. The SAXS data for each concentration were investigated for aggregation using Guinier plots. Radius of gyration estimates were derived by the Guinier approximation $I(q) = I(0) \exp(-q^2R_g^2/3)$ with $qR_g < 1.3$, where $q = 4\pi\sin\theta/\lambda$ and were evaluated in PRIMUS. GNOM (Svergun, 1992) was used to compute the pair distribution function, $P(r)$, and to determine the maximum particle dimension. Chicken egg white lysozyme was used as a protein standard to estimate the molecular weight from $I(0)$ calculated by GNOM. A slight increase in particle R_g was detected as a function of concentration when analysing all three dilutions of the protein solution, suggesting the presence of a larger oligomer. Evaluation of various symmetry mates coupled with the use of the software program OLIGOMER identified a candidate for the larger oligomer as a dimer of the crystallographically determined complex of PanD(T57V)-PanZ. OLIGOMER estimated a volume fraction of 32% of the larger oligomer in the lowest concentration and 39% in the highest concentration. Therefore, a 39% contribution of the simulated scattering profile of the larger oligomer, computed by FoXS (Schneidman-Duhovny et al., 2013), was subtracted from the SAXS data of the highest concentration to yield the scattering from a single PanD(T57V)-PanZ complex molecule for further analysis. Ten *ab initio* shape reconstructions were generated by DAMMIF (Franke and Svergun, 2009) using P4 symmetry

and averaged with DAMAVER (Volkov and Svergun, 2003). CORAL (Petoukhov et al., 2012) was used determine the positions of the crystallographically disordered residues. SUPCOMB (Kozin and Svergun, 2001) was used to align the high-resolution model with the envelope reconstruction).

Small molecule NMR

Activated ADC was obtained via enzymatic activation. A 2:1 mixture of PanD and PanZ (final concentrations 15.8 and 31.7 μM respectively) in 50 mM Tris–HCl, 100 mM NaCl, 0.1 mM DTT, pH 7.4 with 50 μM AcCoA was incubated for 16 hours at 37 °C. The mixture was diluted into 50 mM K_2HPO_4, 100 mM NaCl, pH 7.4 to a final working concentration of 100 nM ADC and 200 nM PanZ (and consequently 0.32 μM AcCoA). Incubation samples were supplemented with AcCoA, PanZ and L-aspartate as indicated. ^{1}H NMR samples were made to 10% D_2O (630 μL reaction mixture with 70 μL D_2O) and the 1D ^{1}H NMR spectrum recorded immediately using a water suppression pulse sequence. Product formation was monitored by analysis of the ratio between the aspartate CαH and the β-alanine CH_2 signals determined by integration using Mestrenova (Mestrelab research software).

Emsley, P., Lohkamp, B., Scott, W.G., and Cowtan, K. (2010). Features and development of Coot. Acta Cryst. *D66*, 486-501.

Evans, P.R., and Murshudov, G.N. (2013). How good are my data and what is the resolution? Acta crystallographica. Section D, Biological crystallography *69*, 1204-1214.

Franke, D., and Svergun, D.I. (2009). DAMMIF, a program for rapid ab-initio shape determination in small-angle scattering. J. Appl. Cryst. *42*, 342-346.

Gasteiger, E., Hoogland, C., Gattiker, A., Duvaud, S.e., Wilkins, M., Appel, R., and Bairoch, A. (2005). Protein Identification and Analysis Tools on the ExPASy Server. In The Proteomics Protocols Handbook, J. Walker, ed. (Humana Press), pp. 571-607.

Houtman, J.C., Brown, P.H., Bowden, B., Yamaguchi, H., Appella, E., Samelson, L.E., and Schuck, P. (2007). Studying multisite binary and ternary protein interactions by global analysis of isothermal titration calorimetry data in SEDPHAT: application to adaptor protein complexes in cell signaling. Protein science : a publication of the Protein Society *16*, 30-42.

Keller, S., Vargas, C., Zhao, H., Piszczek, G., Brautigam, C.A., and Schuck, P. (2012). High-precision isothermal titration calorimetry with automated peak-shape analysis. Analytical chemistry *84*, 5066-5073.

Konarev, P.V., Volkov, V.V., Sokolova, A.V., Koch, M.H.J., and Svergun, D.I. (2003). PRIMUS: a Windows PC-based system for small-angle scattering data analysis. J. Appl. Cryst. *36*, 1277-1282.

Kozin, M.B., and Svergun, D.I. (2001). Automated matching of high- and low-resolution structural models. J. Appl. Cryst. *34*, 33-41.

Leslie, A.W., and Powell, H. (2007). Processing diffraction data with mosflm. In Evolving Methods for Macromolecular Crystallography, R. Read, and J. Sussman, eds. (Springer Netherlands), pp. 41-51.

Lovell, S.C., Davis, I.W., Arendall, W.B., 3rd, de Bakker, P.I., Word, J.M., Prisant, M.G., Richardson, J.S., and Richardson, D.C. (2003). Structure validation by Calpha geometry: phi,psi and Cbeta deviation. Proteins *50*, 437-450.

Monteiro, D.C., Rugen, M.D., Shepherd, D., Nozaki, S., Niki, H., and Webb, M.E. (2012). Formation of a heterooctameric complex between aspartate alpha-decarboxylase and its cognate activating factor, PanZ, is CoA-dependent. Biochem. Biophys. Res. Commun. *426*, 350-355.

Murshudov, G.N., Skubak, P., Lebedev, A.A., Pannu, N.S., Steiner, R.A., Nicholls, R.A., Winn, M.D., Long, F., and Vagin, A.A. (2011). REFMAC5 for the refinement of macromolecular crystal structures. Acta Cryst. *D67*, 355-367.

Nozaki, S., Webb, M.E., and Niki, H. (2012). An activator for pyruvoyl-dependent l-aspartate α-decarboxylase is conserved in a small group of the γ-proteobacteria including Escherichia coli. MicrobiologyOpen *1*, 298-310.

Petoukhov, M.V., Franke, D., Shkumatov, A.V., Tria, G., Kikhney, A.G., Gajda, M., Gorba, C., Mertens, H.D.T., Konarev, P.V., and Svergun, D.I. (2012). New developments in the ATSAS program package for small-angle scattering data analysis. J. Appl. Cryst. *45*, 342-350.

Saldanha, S.A., Birch, L.M., Webb, M.E., Nabbs, B.K., von Delft, F., Smith, A.G., and Abell, C. (2001). Identification of Tyr58 as the proton donor in the aspartate alpha-decarboxylase reaction. Chem. Commun. *18*, 1760-1761.
Schmitzberger, F., Kilkenny, M.L., Lobley, C.M., Webb, M.E., Matak-Vinkovic, D., Witty, M., Chirgadze, D.Y., Smith, A.G., Abell, C., and Blundell, T. (2003). Structural constraints on protein self-processing in L-aspartate-alpha-decarboxylase. EMBO J. *22*, 6193-6204.
Schneidman-Duhovny, D., Hammel, M., Tainer, J.A., and Sali, A. (2013). Accurate SAXS Profile Computation and its Assessment by Contrast Variation Experiments. Biophys. J. *105*, 962-974.
Studier, F.W. (2005). Protein production by auto-induction in high-density shaking cultures. Prot. Exp. Purif. *41*, 207-234.
Svergun, D. (1992). Determination of the regularization parameter in indirect-transform methods using perceptual criteria. J. Appl. Cryst. *25*, 495-503.
Vagin, A., and Teplyakov, A. (1997). MOLREP: an Automated Program for Molecular Replacement. J. Appl. Cryst. *30*, 1022-1025.
Volkov, V.V., and Svergun, D.I. (2003). Uniqueness of ab initio shape determination in small-angle scattering. J. Appl. Cryst. *36*, 860-864.
Webb, M.E., Yorke, B.A., Kershaw, T., Lovelock, S., Lobley, C.M.C., Kilkenny, M.L., Smith, A.G., Blundell, T.L., Pearson, A.R., and Abell, C. (2014). Threonine 57 is required for the post-translational activation of E. coli aspartate a-decarboxylase. Acta Cryst. *D70*, 1166-1172.
